# Engineering single topological π-conjugated polymers and interpolating topological solitons by end-group modification

**DOI:** 10.1093/nsr/nwag144

**Published:** 2026-03-09

**Authors:** Zhengya Wang, Yunan Li, Bin Li, Jianing Wang, Yingying Wu, Ruoting Yin, Jufeng Wang, Xinyong Meng, Yifan Liang, Xiaoqing Wang, Qing-Song Deng, Yuan-Zhi Tan, Qitang Fan, Chuanxu Ma, Shijing Tan, Qunxiang Li, Jinlong Yang, Bing Wang

**Affiliations:** Hefei National Research Center for Physical Sciences at the Microscale, CAS Center for Excellence in Quantum Information and Quantum Physics, and New Cornerstone Science Laboratory, University of Science and Technology of China, Hefei 230026, China; Hefei National Laboratory, Hefei 230088, China; Hefei National Research Center for Physical Sciences at the Microscale, CAS Center for Excellence in Quantum Information and Quantum Physics, and New Cornerstone Science Laboratory, University of Science and Technology of China, Hefei 230026, China; Hefei National Laboratory, Hefei 230088, China; Hefei National Research Center for Physical Sciences at the Microscale, CAS Center for Excellence in Quantum Information and Quantum Physics, and New Cornerstone Science Laboratory, University of Science and Technology of China, Hefei 230026, China; Hefei National Laboratory, Hefei 230088, China; Hefei National Research Center for Physical Sciences at the Microscale, CAS Center for Excellence in Quantum Information and Quantum Physics, and New Cornerstone Science Laboratory, University of Science and Technology of China, Hefei 230026, China; Hefei National Laboratory, Hefei 230088, China; Hefei National Research Center for Physical Sciences at the Microscale, CAS Center for Excellence in Quantum Information and Quantum Physics, and New Cornerstone Science Laboratory, University of Science and Technology of China, Hefei 230026, China; Hefei National Laboratory, Hefei 230088, China; Hefei National Research Center for Physical Sciences at the Microscale, CAS Center for Excellence in Quantum Information and Quantum Physics, and New Cornerstone Science Laboratory, University of Science and Technology of China, Hefei 230026, China; Hefei National Laboratory, Hefei 230088, China; Hefei National Research Center for Physical Sciences at the Microscale, CAS Center for Excellence in Quantum Information and Quantum Physics, and New Cornerstone Science Laboratory, University of Science and Technology of China, Hefei 230026, China; Hefei National Laboratory, Hefei 230088, China; Hefei National Research Center for Physical Sciences at the Microscale, CAS Center for Excellence in Quantum Information and Quantum Physics, and New Cornerstone Science Laboratory, University of Science and Technology of China, Hefei 230026, China; Hefei National Laboratory, Hefei 230088, China; Hefei National Research Center for Physical Sciences at the Microscale, CAS Center for Excellence in Quantum Information and Quantum Physics, and New Cornerstone Science Laboratory, University of Science and Technology of China, Hefei 230026, China; Hefei National Laboratory, Hefei 230088, China; Hefei National Research Center for Physical Sciences at the Microscale, CAS Center for Excellence in Quantum Information and Quantum Physics, and New Cornerstone Science Laboratory, University of Science and Technology of China, Hefei 230026, China; Hefei National Laboratory, Hefei 230088, China; Collaborative Innovation Center of Chemistry for Energy Materials, State Key Laboratory for Physical Chemistry of Solid Surfaces, and Department of Chemistry, College of Chemistry and Chemical Engineering, Xiamen University, Xiamen 361005, China; Collaborative Innovation Center of Chemistry for Energy Materials, State Key Laboratory for Physical Chemistry of Solid Surfaces, and Department of Chemistry, College of Chemistry and Chemical Engineering, Xiamen University, Xiamen 361005, China; Hefei National Research Center for Physical Sciences at the Microscale, CAS Center for Excellence in Quantum Information and Quantum Physics, and New Cornerstone Science Laboratory, University of Science and Technology of China, Hefei 230026, China; Hefei National Laboratory, Hefei 230088, China; Hefei National Research Center for Physical Sciences at the Microscale, CAS Center for Excellence in Quantum Information and Quantum Physics, and New Cornerstone Science Laboratory, University of Science and Technology of China, Hefei 230026, China; Hefei National Laboratory, Hefei 230088, China; Hefei National Research Center for Physical Sciences at the Microscale, CAS Center for Excellence in Quantum Information and Quantum Physics, and New Cornerstone Science Laboratory, University of Science and Technology of China, Hefei 230026, China; Hefei National Laboratory, Hefei 230088, China; Hefei National Research Center for Physical Sciences at the Microscale, CAS Center for Excellence in Quantum Information and Quantum Physics, and New Cornerstone Science Laboratory, University of Science and Technology of China, Hefei 230026, China; Hefei National Laboratory, Hefei 230088, China; Hefei National Research Center for Physical Sciences at the Microscale, CAS Center for Excellence in Quantum Information and Quantum Physics, and New Cornerstone Science Laboratory, University of Science and Technology of China, Hefei 230026, China; Hefei National Laboratory, Hefei 230088, China; Hefei National Research Center for Physical Sciences at the Microscale, CAS Center for Excellence in Quantum Information and Quantum Physics, and New Cornerstone Science Laboratory, University of Science and Technology of China, Hefei 230026, China; Hefei National Laboratory, Hefei 230088, China

**Keywords:** π-conjugated polymers, topological phases, topological solitons, on-surface synthesis, scanning probe microscopy

## Abstract

Topological solitons can act as mobile domain walls between topologically non-trivial and trivial phases, merging hybrid zero-mode properties from both solitonic and symmetry-protected boundary states, and providing both fundamental insights and unprecedented opportunities for quantum technologies. However, their experimental realization is challenging. Here, we demonstrate on-surface engineering of topological structures and introduction of topological solitons in π-conjugated pentacene polymers through end-group modification on Au(111), using combined multiple techniques including scanning tunneling microscopy, non-contact atomic force microscopy and tip-enhanced Raman spectroscopy, along with density functional theory and tight-binding calculations. We fabricate cumulene-bridged pentacene oligomers and polymers with nearly length independence by anchoring both their termini to the surface. By converting a near-end segment into the trivial phase through its end-group modification, we realize the interpolation of topological solitons as domain walls between non-trivial and trivial phases, which are well supported by observations of the solitonic zero-energy peaks across the domain walls, the band reverse between the separated regions, and the distinct region-dependent vibration modes, as well as theoretical calculations. The realization of topological solitons as domain walls between non-trivial and trivial phases offers a rich platform for fundamental research, and illustrates potential applications of π-conjugated polymers in quantum devices.

## Teaser

This work reports the realization of topological solitons as domain walls between nontrivial and trivial phases in π-conjugated pentacene polymers, merging hybrid zero-modes from both solitonic and topological end states.

## INTRODUCTION

The field of organic topological insulators (OTIs) [1] lies at the intersection of condensed matter physics [2–4], chemistry [5–8] and materials science [9–12], offering abundant opportunities for exploring new physics and developing innovative technologies [13–16]. The ability to create designer OTIs through chemical synthesis makes this a promising research area [17,18]. However, developing and realizing OTIs across various dimensions remains significantly more challenging than for their inorganic counterparts. For 1D OTIs, such as *π*-conjugated polymers—arguably the simplest prototypical system—various strategies have been proposed to engineer the topological properties through introducing strain [19–21], applying electric fields [22,23] or gating [24,25], as well as transport measurements [26], but these approaches prove technically challenging in addressing their topological phases of individual polymers. Very recently, topological phase transitions have been observed in on-surface synthesized conjugated polymers [27–30] and graphene nanoribbons [16,31,32], in which the topological phase transitions occur at a critical length or width due to the enhanced quantum fluctuations [33] as the quasi-1D systems increase in size. Meanwhile, the 1D topologically non-trivial phases have been realized in inorganic materials [10,11], and also have been simulated using quantum matter of ultracold atoms [2,34]. Despite these advances, an effective method for controlling 1D topological phases remains elusive.

Among the various topological phenomena, 1D topological systems hold particular significance as they provide the simplest yet profoundly illustrative platforms for exploring fundamental topological concepts [3,4,35,36]. Similar to their higher-dimensional counterparts [3], 1D OTIs (or TIs) feature an insulating bulk gap and characteristic zero-energy end states localized at their terminals. These 1D topologically non-trivial phases are protected by the chiral symmetry (also known as sublattice symmetry), particle–hole symmetry (or charge-conjugation symmetry) and time-reversal symmetry, belonging to the BDI class [4,37,38]. The Su–Schrieffer–Heeger (SSH) model [5,39] describes these non-trivial phases, which are classified by the Zak phase [40] and the bulk winding number [36]. Within the SSH model’s tight-binding (TB) approximation, 1D topological properties emerge from a dimerized chain with alternating single (weak) and double (strong) bonds—a structure resembling *trans*-polyacetylene. The Zak phase, quantized at either π or 0, corresponding to the topological invariant *Z*_2_ = 1 or 0, determines whether the system is in a non-trivial or trivial phase. Crucially, this topological distinction depends on where the chain terminates [34,36]; cutting at a double bond yields a non-trivial phase with topological end states, while cutting at a single bond results in a trivial phase without end states. Despite the conceptual elegance of the SSH model, practical realization of topological phases in polymeric systems faces significant challenges. Notably, topological phase transitions in polymers have been observed to occur only when the chain length exceeds a critical threshold, even when suitable terminal groups are employed [27–29,32]. This length-dependent behavior imposes fundamental constraints on the on-demand manipulation and tunability of topological phases, presenting a considerable obstacle for the design of compact topological devices. Addressing these limitations requires innovative strategies that can decouple topological phase transitions from stringent length requirements while maintaining robust topological protection.

**Figure 1.**
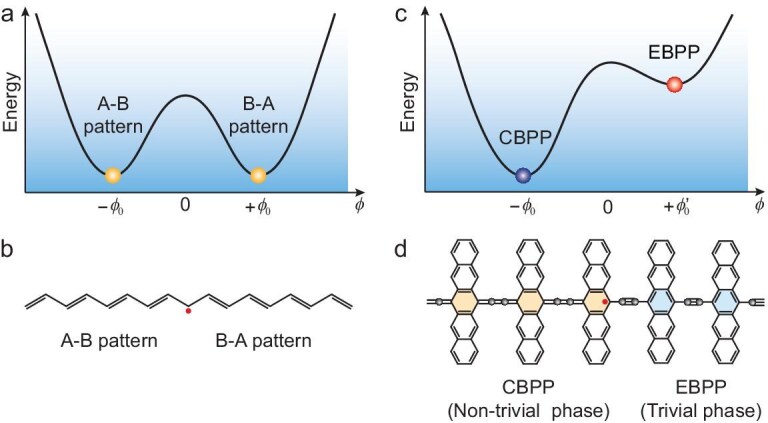
Energy diagrams in the presence of topological solitons. (a) Total energy as a function of order parameter *ϕ* in *trans*-polyacetylene. The twofold-degenerate ground states at ±*ϕ*_0_ associate with the opposite alternating dimerization pattern due to spontaneous symmetry breaking from a hypothetical equal bond length. (b) The Su-Schrieffer-Heeger (SSH) model of a topological soliton between regions with different sublattice A-B and B-A patterns of the degenerate ground states. (c) Total energy as a function of order parameter *ϕ* in pentacene polymer. The non-degenerate energy minima associate with distinct non-trivial and trivial phases. (d) Schematic drawing of a topological soliton separating non-trivial and trivial phase regions in different configurations of pentacene polymer. CBPP and EBPP show the cumulene-quinoid and ethynylene-aromatic features along the backbone, respectively. In these polymers, the bond length alternation (BLA) can be approximately used as the order parameter, *ϕ* = BLA.

On the other hand, the seminal work based on the SSH model has led to the groundbreaking discovery that mobile topological solitons serve as the primary charge carriers responsible for the high conductivity of doped polymers [5,39,41]. Topological solitons have attracted considerable attention because of their interesting properties [41], such as high conductivity [41–43], spin–charge separation [44,45] and fractional charges [44–46]. Remarkably, topological solitons and topological end states are fundamentally distinct classes of topological excitations, but they are sometimes confused in the literature due to being closely related. Topological solitons are non-linear excitations that arise from spontaneous symmetry breaking and are protected by the topology of the order parameter space in field theories [47,48]. In fact, in the initial SSH model, topological solitons that emerge as mobile domain walls can exist without requiring spatially separated regions with specific topological phases, such as the opposite alternating dimerization patterns in *trans*-polyacetylene [5,39] (Fig. 1a and b). However, it is particularly compelling to investigate scenarios where topological solitons play a more sophisticated role—specifically as domain walls that separate regions with distinct topological phases (Fig. 1c and d). 1D OTIs would potentially combine the mobile nature of solitonic excitations with the robust properties of topological phase transitions, yet this has not been experimentally achieved.

In this work, we demonstrate an approach for engineering topological structures and interpolating topological solitons in π-conjugated polymers through end-group modification. We achieve the precise synthesis of pentacene polymers with both their termini anchored on the Au(111) surface using optimized on-surface synthesis conditions similar to those employed for acene polymer preparation [27,28,49]. Using a combination of tip-based techniques [50,51], including scanning tunneling microscopy (STM), non-contact atomic force microscopy (nc-AFM) and tip-enhanced Raman spectroscopy (TERS), along with density functional theory (DFT) and TB calculations, we identify that the pentacene polymers are present as cumulene-bridged polypentacene (CBPP) regardless of their lengths. We confirm that CBPPs exhibit a topological structure with cumulene-quinoid *π*-conjugation, and demonstrate that a topological soliton can be introduced to separate non-trivial and trivial phase regions by converting a polymer segment to the trivial phase through end-group modification.

## RESULTS

### Cumulene-bridged pentacene species formed by anchoring both termini to Au(111) surface

**Figure 2.**
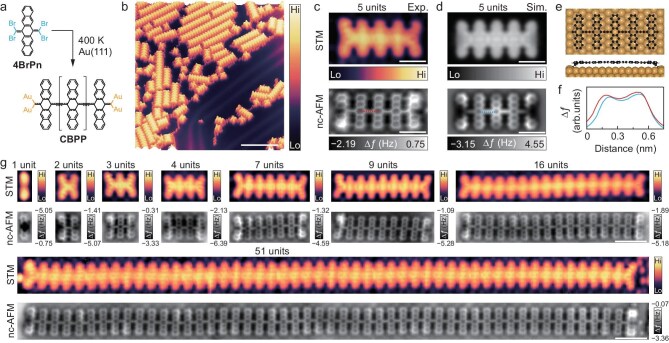
Structural characterization of on-surface synthesized cumulene-bridged pentacene oligomers and polymers. (a) Scheme for synthesis of cumulene-bridged pentacene oligomers and polymers on Au(111) using precursor molecules of 4BrPn. (b) Large-area STM image of formed pentacene oligomers and polymers on Au(111). (c) Experimental STM image (upper panel) and nc-AFM image (lower panel) of a cumulene-bridged pentacene pentamer (with five repeating units). (d) Simulated STM image at 1.0 V with Gaussian smoothing (upper panel) and nc-AFM image (lower panel). (e) Optimized structural model in top and side views, respectively. (f) Line profiles measuring the cumulene-like bridging bond along the marked red and blue dotted lines in the experimental and simulated nc-AFM images of (c) and (d). (g) A set of paired STM and nc-AFM images in upper and lower panels for cumulene-bridged pentacene oligomers or polymers with different lengths, labelled with the number of repeating units, from *n* = 1 up to *n* = 51. Both STM and nc-AFM images were acquired using a CO-functionalized tip. STM imaging parameters: sample bias *V*_s_ = 1.0 V and tunneling current *I*_t_ = 30 pA in (b); *V*_s_ = 1.0 V and *I*_t_ = 10 pA in (c); *V*_s_ = 1.0 V and *I*_t_ = 50 pA in (g) for *n* = 1, 2, 3, 4, 7, 16 and 51 units, but *V*_s_ = 1.0 V and *I*_t_ = 10 pA for *n* = 9. AFM imaging parameters: quality factor *Q* ∼ 5000. During nc-AFM imaging at constant-height mode, a sample bias *V*_s_ = 10 mV was applied for taking current images simultaneously (as given in Fig. 3 below). All experiments were performed at 5 K. Scale bars: 6 nm in (b), 1 nm in (c) and (d), 2 nm in (g).

Using the precursor molecule 6,13-bis(dibromo methylene)-6,13-dihydropentacene (4BrPn), and through optimizing the on-surface synthesis conditions employed in the preparation of acene polymers [27,28,49], we achieved the synthesis of pentacene oligomers or polymers on Au(111) at an annealing temperature of 400 K (Fig. 2a, b and Fig. S1). This relatively low annealing temperature can be beneficial for preventing the removal of the end carbene-like C atoms, different from those at the higher annealing temperature of 500 K [27,28]. Figure 2c shows typical STM and nc-AFM images of a pentacene pentamer (with five repeating units, *n* = 5). The experimental images are well reproduced by the simulated STM and nc-AFM images (Fig. 2d), according to the optimized configuration by considering both-side carbene-like terminal C atoms that are anchored to the Au surface atoms (Fig. 2e). In this optimized structure, each of the terminal C atoms mainly locates at the hollow site formed by three neighboring surface Au atoms, with two C–Au bond distances slightly shorter by 0.12 Å than the third. This oligomer or polymer terminal configuration is also schematically illustrated in Fig. 2a, where each carbene-like terminal is stabilized by two Au atoms on both sides. The bond-resolved nc-AFM image along with the simulation shows that the outermost pentacene moieties near the termini are highly tilted compared to the inner ones, supporting the suggestion that the terminal C atoms are anchored to the surface Au atoms. Our recent work shows that other types of termini without anchoring to the surface may also exist [49], such as terminated by C–CH_3_, which leads to the outermost pentacene moieties tilting slightly.

Another significant feature is the absence of the ethynylene-like (–C≡C–) signature in the short species with only a few repeating units. This is more clearly shown in the line profiles from both the experimental and simulated nc-AFM images (Fig. 2f), also showing independence on varied tip-sample distances (Fig. S2). It is noted that the ethynylene-like bonds typically exhibit a relatively protruded spot [27,28,49], existing in ethynylene-bridged pentacene polymers (EBPPs) as a topologically trivial phase, such as the observed pentacene polymers shorter than 26 repeating units on Au(111) [28]. This spot-like signature’s absence strongly indicates that the inter-pentacene bridging bonds possess increased cumulene-like (=C=C=) character, which corresponds to the bridging bond configuration in long acene polymers after undergoing topological phase transition into their non-trivial phase [27,28].

Figure 2g shows a set of STM and corresponding nc-AFM images of pentacene oligomers or polymers containing different repeating-unit numbers, from monomer (*n* = 1) to polymers with *n* = 51. All these species show the feature of the both termini anchored to the surface, including the monomer that contains only one unit. Starting from the dimer (*n* = 2), the bridging bonds in the nc-AFM images all present a uniform feature, significantly different from the ethynylene-like bonds with a spot-like protrusion. Their corresponding STM images also present overall uniform topography. These observations suggest that these pentacene oligomers or polymers with both termini anchored to the surface most likely already present as cumulene-like bridging bonds between pentacene moieties. As reported in our recent work [49], the Raman spectra taken at bridging bonds of a pentacene polymer with anchored termini show a uniform cumulene-like character with nearly unchanged lower frequency at 2002 cm^−1^, compared to the ethynylene-like bonds with higher frequency at around 2040 cm^−1^. If not specified, we use the term CBPP to describe cumulene-bridged pentacene species with *n* ≥ 5, though this nomenclature is applied loosely.

The observation of these cumulene-bridged pentacene oligomers and CBPPs is remarkable, raising the intriguing question of whether these CBPPs possess topological non-triviality. Indeed, previous studies on acene polymers synthesized at 500 K have reported cumulene-bridging bonds appearing in the topologically non-trivial phase during critical topological phase transitions [27,28]. Specifically, in pentacene polymers, a transformation from the topologically trivial EBPP phase to the non-trivial CBPP phase is observed at a critical threshold of 26 or 15 repeating units, depending on the molecular coverage, accompanied by the emergence of end states [28,29]. Notably, in those systems, both termini are located at the acene edge because the removal of carbene-like C atoms prevents surface anchoring—a key distinction from our current observations.

### Electronic behaviors of cumulene-bridged pentacene polymers

**Figure 3.**
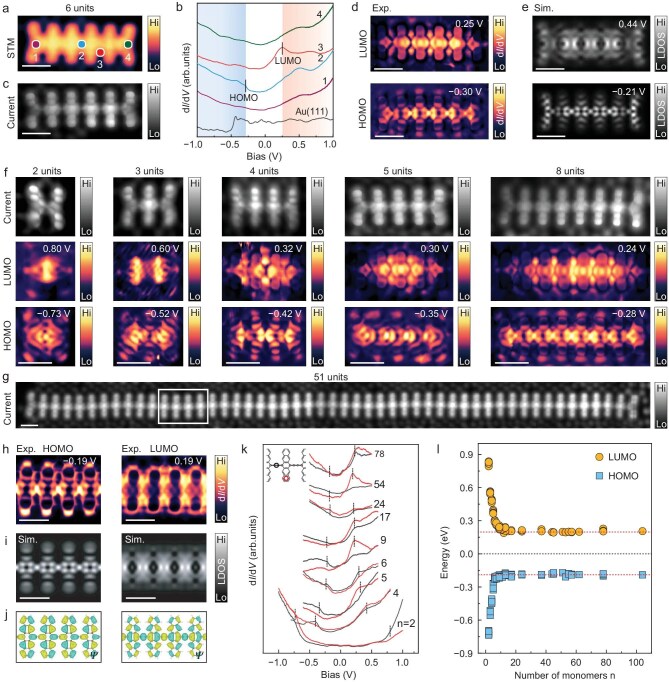
Electronic properties of cumulene-bridged pentacene oligomers and polymers. (a) STM topographic image of a hexamer (six repeating units), taken at *V*_s_ = 1.0 V, *I*_t_ = 50 pA. (b) d*I*/d*V* spectra (*V*_s_ = 1.0 V, *I*_t_ = 50 pA, modulation *V*_rms_ = 10 mV) taken at the marked site in (a), with a reference spectrum from bare Au surface, vertically shifted for clarity. (c) Constant-height current image at a bias voltage of 10 mV (a near-zero bias) within the same area as in (a). (d) d*I*/d*V* maps recorded at the energies of LUMO (at 0.25 eV) and HOMO (at −0.30 eV). (e) DFT-simulated LDOS distributions of the LUMO and HOMO. (f) A set of cumulene-bridged pentacene oligomers with different lengths, showing their constant-height current images (upper panels) and their distributions of the LUMO (middle panels) and HOMO (lower panels). (g) Constant-height current image of a CBPP with repeating-unit number *n* = 51, at a near-zero bias of 10 mV. (h) d*I*/d*V* maps taken at the energies of LUMO (at 0.19 eV) and HOMO (at −0.19 eV) within the marked segment in (g). (i) DFT-simulated LDOS maps and (j) wavefunctions corresponding to HOMO (left) and LUMO (right) in freestanding infinite pentacene polymer. In (e) and (i), a tip condition that contains 85% *p*-orbital and 15% *s*-orbital was adopted to mimic the behavior of the CO-functionalized tip. In (j), cyan and yellow denote different signs of wavefunctions. (k) Collection of typical d*I*/d*V* spectra from oligomers or polymers with labeled repeating-unit number *n*, vertically shifted for clarity, with the short vertical dashed lines marking the peak/step-like features corresponding to HOMO and LUMO. (l) Plot of positions of HOMO and LUMO, extracted from Fig. S4a, as a function of *n*. Each point gives the typical results of a polymer with a given length. The experimental d*I*/d*V* maps were acquired at setpoint conditions of the labeled biases in each panel, and *I*_t_ = 50 pA, using lock-in amplifier with *V*_rms_ = 10 mV. All the measurements were performed using a CO-functionalized tip at 5 K. Scale bars: 1 nm.

We now characterize the electronic properties of these pentacene oligomers or polymers across different lengths, all with both termini anchored to the Au surface. Using a hexamer (with six repeating units) as an example (Fig. 3a), the typical d*I*/d*V* spectra taken within bulk sites show a characteristic semiconducting behavior, with the highest occupied molecular orbital (HOMO) and lowest unoccupied molecular orbital (LUMO) at −0.30 and 0.25 eV (Fig. 3b), respectively. The near-zero-bias constant-height current image (Fig. 3c) shows a relatively uniform feature for the bulk bonds, and a less pronounced feature at both termini. The less pronounced feature is consistent with the anchored termini revealed by the nc-AFM images above. No zero-energy end state is observed in the d*I*/d*V* spectra at the termini (Fig. 3b). The d*I*/d*V* maps of the LUMO and HOMO also show relatively uniform distributions mainly along the polymer backbone (Fig. 3d), which are well reproduced by the DFT simulated maps of the local densities of states (LDOS) from the LUMO and HOMO (Fig. 3e), respectively. Both experimental and calculated results demonstrate that the characteristic features do not depend on whether the number of repeating units is odd or even (Fig. S3).

The typical structures taken at near-zero bias (typically at 10 mV) and electronic distributions of the LUMO and HOMO exhibit similar patterns across various oligomers (Fig. 3f) and the CBPP with *n* = 51 (Fig. 3g and h), consistent with the observations in the hexamer above. With *n* ≥ 5, it is observed that the LUMO and HOMO distributions are very similar to the ones observed in the CBPP with *n* = 51 (Fig. 3h), where the LUMO and HOMO distributions are taken from the marked segment in Fig. 3g. The simulated LDOS distributions using CBPP crystal by DFT (Fig. 3i and j) well reproduce the main features in Fig. 3h.

The revealed HOMO and LUMO through the combined d*I*/d*V* spectra and maps show that the energy positions of HOMO and LUMO reduce quickly from *n* = 2 to *n* = 8, which are almost symmetrically presented relative to the Fermi level (Fig. 3f and k), suggesting the negligible doping. For *n* ≥ 9, HOMO and LUMO keep almost unchanged, which can be further verified by representative d*I*/d*V* maps at the assigned orbital positions (Fig. S4), although the intensities of HOMO are relatively weak compared to those of LUMO. Our Bader analysis confirms that the anchoring of both termini to the surface leads to negligible charge transfer, for example, about 0.04 electrons per repeating unit from the hexamer to the surface, much smaller than the value of 0.20 electrons per repeating unit in the presence of both CH_3_ termini instead with three H atoms bonded to each terminal carbene-like C atom (Fig. S5), as observed in our recent work [49]. The calculated changes in bond length and bond length alternation (BLA) suggest that negligible doping is critical to keep the cumulene-like bridging bonds by suppressing the Peierls instability, whereas the presence of both CH_3_ termini leads to relatively large BLA variations due to enhanced doping, in line with the fact that charging can usually enhance the electron–phonon coupling and lead to the Peierls distortions, as observed in doping-induced EBPPs [49]. Our calculations strongly indicate that anchoring both termini to the surface preserves the cumulene-like bridging bonds, suggesting the formation of CBPP rather than EBPP. These theoretical results corroborate the experimental observations of CBPPs described above.

### Topological properties of cumulene-bridged pentacene polymers

Previously, the topological phase transition from a trivial EBPP phase to a non-trivial CBPP phase was observed with the increase of the polymer length [28,29], where the end states can be observed near the Fermi level when the system enters the non-trivial phase. This non-trivial topology arises from the cumulene-quinoid character along the backbone. Our DFT and TB calculations reveal that the CBPP crystal (infinitely long CBPP) can be topologically trivial or non-trivial, corresponding to the topological invariant *Z*_2_ = 0 and 1, depending on the choice of the unit cell or practically determined by the termination (Figs S6 and S7). While the anchored termini indeed play a key role in preserving the observed CBPPs, the effects of these termini can also be significant on the behaviors of the end states [52]. However, it is difficult to directly analyze a CBPP with both anchored termini on the Au(111) surface. Instead, we consider a relatively simple system of freestanding pentacene polymers with CH_2_ or CH_3_ termini on both sides (Figs S8 and S9), analogous to the possible effects of the anchored CBPP termini on Au(111). The effects of CH_2_ and CH_3_ termini on bond length and BLA show distinct behaviors as a function of polymer length *n* (Fig. S8). The CH_2_-terminated pentacene polymers exhibit cumulene-like bridging bonds at all lengths, including the dimer, as evidenced by both the critical bond length and BLA at which the topological phase transition occurs (as defined in Fig. S7). In contrast, for CH_3_-terminated pentacene polymers, the topological phase transition is expected to occur only when the polymer length exceeds the critical value of approximately *n* = 4 (Fig. S9), although the DFT calculations may underestimate the critical length [28]. Notably, there are no near-Fermi-level end states within the bulk HOMO–LUMO gap in the CH_2_-terminated pentacene polymers (Fig. S9a and b). While there indeed exist the non-zero-energy end states around ±1.2 eV, they generally locate at the end pentacene moiety and the CH_2_ group, exhibiting the topologically trivial nature. Accordingly, despite examining all these cumulene-bridged oligomers and polymers with the Au-anchored termination, we did not experimentally observe any features corresponding to the expected zero-energy end states.

Although these simulations with CH_2_ termini may yield different perturbation strengths on chiral symmetry compared to Au-anchored termini, we believe that the underlying physics remains fundamentally similar. This mechanistic similarity suggests that the CBPPs with both anchored termini have an unchanged trivial topology with the consistent cumulene-quinoid features. This result can be understood as follows. In the CBPP with the Au-anchored termination, the terminal carbene-like carbon contributes an additional *p* electron to the π bond, which suggests a unit cell corresponding to the topologically trivial with *Z*_2_ = 0 for the CBPP phase (Fig. S6i). The case is significantly different for the CH_3_ termination, where the terminal carbon is fully saturated with three H atoms and contributes no *p* electron. This in turn results in the unit cell corresponding to the trivial topology with *Z*_2_ = 0 for the EBPP phase (Fig. S6j). The analysis about electronic states and evolvements of gap sizes also gives the consistent results about the topological phase transition in the CH_3_-terminated pentacene polymers, from the trivial EBPP to the non-trivial CBPP (Fig. S9c–e). As also illustrated in Fig. S6b, c, e and f, under the same choice of unit cell, the EBPP and CBPP phases always have opposite topologies with distinct *Z*_2_ values. This points to a manner to deterministically induce the topological soliton state at the EBPP–CBPP domain wall if we can partially convert the CBPP segment to EBPP in a single chain (Fig. S6k and l).

### Interpolating a topological soliton at CBPP–EBPP domain wall

**Figure 4.**
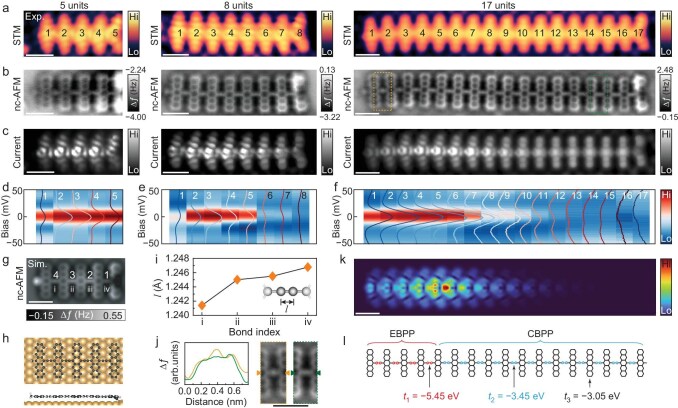
Extended soliton formed at the EBPP–CBPP domain wall in single polymers by end-group modification. (a) STM topographic images, (b) nc-AFM images, and (c) constant-height current images simultaneously recorded during nc-AFM imaging at an applied bias of 10 mV, acquired for polymers with various lengths *n* = 5, 8 and 17. All these images were acquired using a CO-functionalized tip. STM imaging parameters: *V*_s_ = 1.0 V and *I*_t_ = 50 pA. AFM imaging parameters: quality factor *Q ∼* 5000. During nc-AFM imaging at constant-height mode, a sample bias *V*_s_ = 10 mV was applied for taking current images simultaneously (c). (d–f) Waterfall plots of d*I*/d*V* spectra taken at the center of each pentacene unit in the polymers, as labeled in the panels of (a), respectively. d*I*/d*V* measurement parameters: *V*_s_ = −100 mV, *I* = 10 pA, and *V*_rms_ = 3 mV, performed at 5 K. (g) Simulated nc-AFM images of a 5-unit-long polymer with one CH_3_ end (left) and the other end (right) bonded to the Au surface. (h) Optimized structural configuration in top and side views. (i) Calculated lengths of four bridging bonds (i–iv) in (g) and (h). (j) Left panel: line profiles correspondingly along the paired orange and green triangle-marked lines as labeled in the right panel. Right panel: magnified nc-AFM images of the dashed rectangle marked regions in the right panel of (b) near the two terminals, respectively. (k) TB-calculated LDOS map, and (l) the corresponding model, marked with the values of hopping terms for different bonds to simulate the 17-unit-long polymer with an EBPP–CBPP domain wall, corresponding to the right panel of (c). Scale bars: 1 nm.

Our analysis above reveals that pentacene polymers of finite length terminated with CH_3_ groups tend to be the EBPP phase, while termini anchoring to the Au surface present the CBPP phase. This finding provides a pathway to investigate the topological solitons in individual polymers through end-group modification. As shown in Fig. 4 and Fig. S10, this modification is achieved by introducing a small amount of hydrogen to the surface, resulting in an Au-anchored terminus replaced by a CH_3_ terminus. For the polymers with various lengths (Fig. S11), the CH_3_ terminus exhibits distinct features in both topographic STM (Fig. 4a) and nc-AFM images (Fig. 4b). The obviously varied patterns in the near-zero-bias current images (Fig. 4c) can be attributed to the appearance of a zero-energy peak in the d*I*/d*V* spectra taken at these corresponding sites (Fig. S12). For direct comparison, the d*I*/d*V* spectra are given by the waterfall plots along the polymer backbones at the labeled sites in Fig. 4d–f, to show the distributions of the zero-energy peak. Notably, a protrusion appears near the outermost pentacene edge in polymers of various lengths when one terminus is converted to CH_3_ (Fig. 4b). The assignment of the protrusion to a CH_3_ terminus is supported by the simulated nc-AFM image (Fig. 4g), based on the optimized adsorption configuration of a pentamer (*n* = 5) with a CH_3_ terminus on one side and an anchored terminus on the other side (Fig. 4h). Other terminus types, such as CH and CH_2_, do not produce protruding topographic features at the chain end in simulated nc-AFM images (Fig. S13). Bond length analysis reveals an interesting finding that the bridging bond near the anchored terminus maintains a cumulene-like character, while other bonds adopt an ethynylene-like configuration (Fig. 4i, Fig. S14), as classified by bond-length-dependent topological phases (Fig. S7). This is evidenced by the absence or presence of protruding spot-like features at bridging bonds, as highlighted by the magnified nc-AFM images and the corresponding paired line profiles from the opposite near-terminal bonds (Fig. 4j). The observation of spot-like bridging bonds near the CH_3_ terminus directly confirms their conversion to ethynylene-like bridging bonds.

The observed non-uniform patterns in the near-zero bias current images (Fig. 4c) are a result of the presence of the zero-energy peak at domain wall sites between trivial and non-trivial phases along the polymer backbone (Fig. 4d and Fig. S12). These observations represent a significant effect of CH_3_ terminus-induced bond pattern conversion in the nearby polymer region. A zero-energy peak is expected to arise in two distinct contexts: in the field theory, it appears as a mobile topological soliton (alternatively termed a kink or domain wall) [47,48], while in the SSH model, it manifests as a localized end state of a 1D topologically non-trivial phase [5,39]. Interestingly, though the SSH model was initially formulated to describe topological solitons, it has become a standard framework for understanding topological end states. The intersection of these two concepts offers compelling possibilities by merging the mobility of topological solitons with the traditionally fixed nature of topological end states. In this framework, an end state located at a domain wall between trivial and non-trivial phases can be understood as a topological soliton, manifesting their merged hybrid modes. As in *ϕ*^4^ scalar field [47,48], a 1D topological soliton, also known as a kink that is protected by the topology of order parameter space, is one of the most famous examples, where the 2-fold degenerate energy minima of the double-well potential are similar to the ones in *trans*-polyacetylene [5,39] (Fig. 1a). However, as revealed in our calculations (Fig. S7), also schematically illustrated in Fig. 1c, the ethynylene-aromatic and cumulene-quinoid configurations along the polymer backbone possess quite different energies, resulting in non-degenerate energy minima in the double-well potential. The cumulene-quinoid configuration is the ground state, and the ethynylene-aromatic configuration is metastable (Fig. S7). This may explain the observed much shorter critical length of about *n* = 4 for topological phase transition in CH_3_-terminated polymers (Figs S8 and S9).

The solitonic states display a clearly extended feature over 12 units in the 17-unit-long polymer, with the strongest intensity around the fifth unit from the converted end (Fig. 4f). For shorter polymers, the soliton states extend over almost the whole chains (Fig. 4d and e). To effectively simulate the soliton state at the EBPP–CBPP domain wall in the polymer with length *n* = 17, we use the TB model by choosing three different hopping terms to describe the distinct bond orders, as illustrated in Fig. 4l and Fig. S15. Here, *t*_1_ = −5.45 eV for describing the four bridging bonds in the EBPP segment on the left side, *t*_2_ = −3.45 eV for the 12 bridging bonds in the CBPP segment, and *t*_3_ = −3.05 eV for the remaining aromatic bonds, following the previous recipe [27]. The calculated spatial distribution of the zero-mode (Fig. 4k) agrees well with the experimentally observed extended soliton state (right panel in Fig. 4c). This result confirms that the bond order variation in the EBPP–CBPP domain wall is the key to the soliton, consistent with the nc-AFM results.

**Figure 5.**
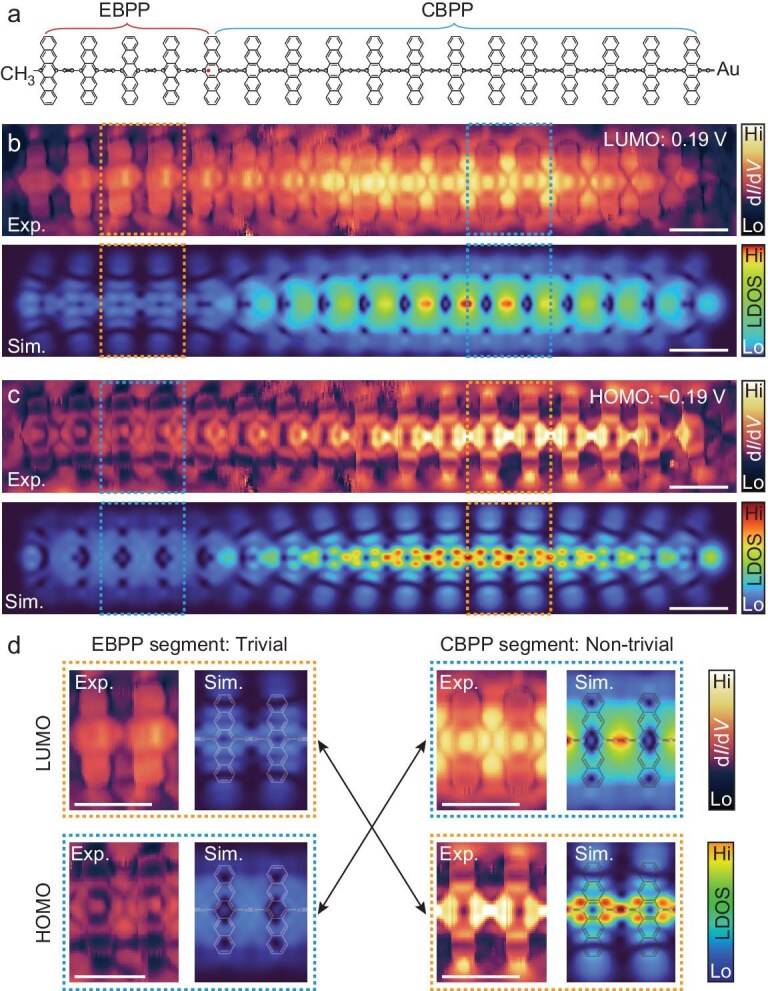
Analysis of HOMO and LUMO in the EBPP and CBPP segments in the single polymer. (a) Chemical sketch of the 17-unit-long polymer with EBPP and CBPP segments, corresponding to the right panel in Fig. 4c. (b and c) Experimental d*I*/d*V* maps (upper) and TB-simulated LDOS maps (lower) of LUMO (b) and HOMO (c). d*I*/d*V* mapping parameters: *I*_t_ = 50 pA and *V*_rms_ = 10 mV. (d) Magnified LUMO (upper) and HOMO (lower) patterns from the left- and right-segments, corresponding to the EBPP and CBPP segments, as marked by the dashed rectangles in (b) and (c). Both the experimental and simulated maps were obtained with a CO-functionalized tip. Scale bars: 1 nm.

The presence of extended solitonic states at the EBPP–CBPP domain walls can be further unraveled by analyzing LUMO and HOMO in distinct phases. Figure 5a schematically shows the EBPP and CBPP separated by the topological soliton according to the patterns in Fig. 4f and k for the polymer (*n* = 17). This can be further confirmed by the measured d*I*/d*V* maps at energies of ±0.19 eV, corresponding to LUMO and HOMO, along with the well reproduced LUMO and HOMO patterns from TB simulations (Fig. 5b and c). It is seen that the LDOS intensities in the longer CBPP segment on the right side are much stronger than those in the left EBPP segment. Distinct patterns between the two segments can be well resolved by the experimental and simulated HOMO and LUMO maps. An obvious band reversal is observed between the EBPP and CBPP segments, which can be more clearly seen by comparing the enlarged patterns in Fig. 5d. This observation gives another piece of evidence for the distinct topological phases of EBPP and CBPP segments separated by the topological soliton.

### Raman spectra of the topological soliton at the CBPP–EBPP domain wall

**Figure 6.**
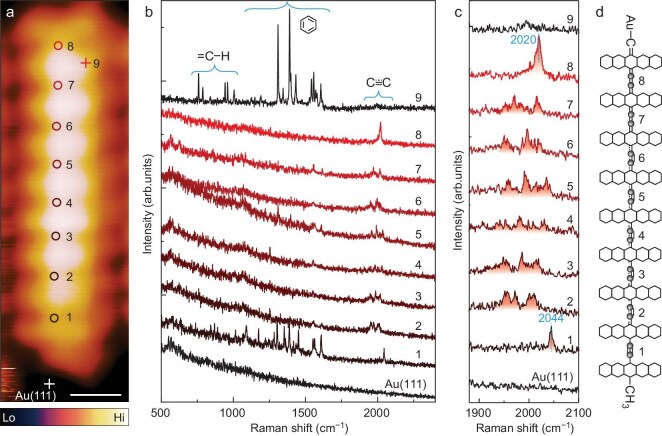
Contact-mode TERS measurements of a polymer containing topological soliton at the EBPP–CBPP domain wall. (a) STM topographic image (*V*_s_ = 0.10 V and *I*_t_ = 100 pA) of a 9-unit-long polymer with the top end bonding to Au and the bottom end converted to CH_3_. Scale bar: 1 nm. (b and c) Same set of Raman spectra, plotted with different frequency ranges, correspondingly obtained at the marked sites 1–9 and on the bare Au(111) surface. TERS measurement parameters for the tip-sample nanocavity: initial tip height set by setpoint of 0.1 V and 8 nA; excitation light: 532 nm and 0.5 mW; CCD spectrometer integration time: 20 s per spectrum; grating: 300 grooves/mm. (d) Chemical sketch of the 9-unit-long polymer with two distinct termini and varied bridging bond orders.

We further measure the local vibrational properties associated with the extended topological soliton across the CBPP–EBPP domain wall using TERS [50,51]. In order to detect the in-plane vibration modes of the ethynylene-like and cumulene-like bonds, we adopt the contact-mode TERS measurements by approaching the tip very close to, and even reaching a point contact [49] to the polymer sites in a polymer (*n* = 9) containing CBPP–EBPP domain wall, as labeled in Fig. 6a. The Raman spectra taken at the marked sites are presented in Fig. 6b with the enlarged Raman shift range from 1900 to 2100 cm^−1^ in Fig. 6c. The bonds corresponding to the measured sites are schematically illustrated in Fig. 6d. Spectrum 9, collected at the pentacene moiety end, shows peaks between 750 and 1000 cm^−1^, corresponding to the out-of-plane vibrations of the peripheral C–H bonds, while those around 1500 cm^−1^ are from the vibrations of benzene rings. Differently, for spectra 1–8 taken at the bridging bonds, there are peaks appearing in the Raman-silent region around 2000 cm^−1^, which are characteristic of the in-plane stretching mode in the ethynylene-like and cumulene-like bonds [49,53]. Variations of Raman peak positions and peak splitting are observed across the EBPP–CBPP domain wall in Fig. 6c. Notably, spectra 1 and 8, taken near the ends of the EBPP and CBPP segments, display single peaks at 2044 and 2020 cm^−1^, respectively, which are similar to the single peak feature at the end sites of a longer (12-unit) polymer containing a CBPP–EBPP domain wall (Fig. S16). The difference in frequency of about 24 cm^−1^ reflects the bond order changes between the ethynylene-like and cumulene-like bonds. There are generally multiple peaks in spectra 2–7, observed in the range approximately from 1954 to 2035 cm^−1^. This result can be reasonably attributed to the broken-symmetry-induced multiple vibration modes associated with the extended topological soliton state, in line with theoretically predicted multiple vibration modes for topological solitons in *trans*-polyacetylene [54,55]. It is noted that near the center of the CBPP–EBPP domain wall, the multiple peaks in spectrum 4 are characterized by relatively weak intensities in a wider frequency range. Our Raman spectra additionally provide vibrational features of the extended topological soliton at a CBPP–EBPP domain wall separating the topologically non-trivial and trivial phases.

## DISCUSSION

While previous observations have reported the topological phase transitions in pentacene-terminated pentacene polymers from ethynylene-like bridging bonds to cumulene-like bridging bonds at the critical lengths of 26 units [27,28] or 15 units [29] depending on the coverage, our work corroborates the deterministic cumulene-bridged pentacene phase in both oligomers and polymers, nearly independent on the length. With both terminals anchored to the Au surface, the achieved cumulene-like phase, even in oligomers as short as dimers, has significant implications. Similar chemical bonding with metal electrodes [56,57] may also happen in polymer- or 2D covalent framework-based electronic devices, where the possible bonding-induced topological structural transitions might be taken into consideration to understand the impressive performances [58,59]. The further local conversion of the segment near the CH_3_ terminal, with the other terminal staying anchored to Au, controllably introduces solitons at the EBPP–CBPP domain wall, even in oligomers with length as short as four repeating units, which is almost impossible for the strategy by utilizing the length-dependent phase transition at a critical length threshold. This advantage holds great potential in few-nanometer and even subnanometer-scale quantum devices based on single topological solitons in short conjugated oligomers/polymers, crucial for post-Moore electronics [60,61].

The spatially extended solitonic states close to the CH_3_ terminals are confirmed by the high-resolution characterizations of the electronic and vibrational properties, which can help to understand prior pioneering studies in this field. In the previous reports of the critical length-dependent topological phase transitions [27–29], the observed topological ‘end’ states do not show the most pronounced intensities exactly at the polymer termini. By considering the obvious protrusions of the ethynylene-like bonds around the terminal units while the inner presented the cumulene-like bonds in their nc-AFM images, one can simply put the observations under the same picture of the formation of the topological soliton states at the EBPP–CBPP domain walls. On the other hand, the state-of-the-art techniques allow us to examine the multiple vibrational modes of the soliton states in individual polymers as predicted 40 years ago [54,55], for the first time at single-bond resolution. These data provide the vibrational fingerprints of the single-bond-scale broken symmetry in the solitons, offering benchmarks for the theoretical modeling of the intertwined structural, electronic and vibrational properties of the solitonic quasiparticles.

## CONCLUSION

We have demonstrated an on-surface approach to engineer the topological structures of π-conjugated pentacene polymers and to create extended topological soliton states through end-group modification. By anchoring both terminals to the Au surface, the suppression of the Peierls instability results in the length-independent CBPP phase with cumulene-like bridging bonds. Converting one terminal into a CH_3_ configuration locally induces a topological phase transition to the trivial EBPP phase with ethynylene-like bridging bonds, leading to the emergence of the topological solitons at the EBPP–CBPP domain walls. By utilizing the joint STM-AFM-TERS characterizations, complemented by the DFT and TB calculations, we unveil the topologically non-trivial electronic properties resulting from the cumulene-quinoid *π*-conjugation in the CBPP phase and the extended electronic and vibrational characteristics of the topological soliton states at the EBPP–CBPP domain walls. The non-trivial topology of the soliton states is further supported by the observed band reversal between the CBPP and EBPP segments. Our findings pave the way for realizing π-conjugated OTIs and topological solitonic quasiparticles, which we envision will contribute to future polymer-based electronics, spintronics and quantum information processing.

## METHODS

Experimental methods are available in the Supplementary data.

## Supplementary Material

nwag144_Supplemental_File
