# Supplementary material for: Engineering single topological π-conjugated polymers and interpolating topological solitons by end-group modification
Source: Natl Sci Rev. 2026 Mar 9;13(7):nwag144. doi: 10.1093/nsr/nwag144 (PMC13070679; doi:10.1093/nsr/nwag144)
Supplement: nwag144_Supplemental_File [file nwag144_supplemental_file.pdf]

Supporting Information for

Engineering single topological π-conjugated polymers and interpolating topological solitons by end-group modification

Zhengya Wang[1,2†], Yunan Li[1,2†], Bin Li [1,2†], Jianing Wang[1,2†],Yingying Wu[1,2], Ruoting Yin[1,2], Jufeng Wang[1,2], Xinyong Meng[1,2], Yifan Liang[1,2], Xiaoqing Wang[1,2], Qing-Song Deng[3], Yuan-Zhi Tan[3], Qitang Fan[1,2], Chuanxu Ma[1,2*], Shijing Tan[1,2], Qunxiang Li[1,2], Jinlong Yang[1,2] and Bing Wang[1,2*]

[1]Hefei National Research Center for Physical Sciences at the Microscale, CAS Center for Excellence in Quantum Information and Quantum Physics, and New Cornerstone Science Laboratory, University of Science and Technology of China, Hefei 230026, China.

[2]Hefei National Laboratory, University of Science and Technology of China, Hefei 230088, China.

[3]Collaborative Innovation Center of Chemistry for Energy Materials, State Key Laboratory for Physical Chemistry of Solid Surfaces, and Department of Chemistry, College of Chemistry and Chemical Engineering, Xiamen University, Xiamen 361005, China.

*Corresponding authors. Email: cxma85@ustc.edu.cn; bwang@ustc.edu.cn.

[†]Equally contributed to this work.

Table of Contents:

Experimental methods

Supporting figures

Experimental methods

On-surface synthesis. The on-surface synthesis was conducted in a preparation chamber equipped with an STM system, operating under ultrahigh vacuum (UHV) conditions with a base pressure of 2×10^{-10} mbar. The Au(111) single crystals used for the experiment was cleaned following a procedure involving repeated cycles of Ar^+ sputtering (1.5 keV, 15 minutes) and annealing (~750 K, 20 minutes) for more than ten cycles. Then, the precursor molecules 6,13-bis(dibromo methylene)-6,13-dihydropentacene (4BrPn) were thermally evaporated from a quartz crucible onto the clean Au(111) surfaces held at room temperature. The evaporation process typically lasted approximately 1.5 minutes. Subsequently, annealing at 400 K for 40 minutes resulted in the formation of pentacene polymers with various length (Fig. S1).

STM-AFM measurements. The STM-AFM measurements were conducted using an Omicron low-temperature microscope equipped with a SPECS Nanonis controller. All STM measurements were carried out at a temperature of 5 K, utilizing electrochemically etched and well-cleaned tungsten (W) tips in a constant-current mode. A bias voltage was applied to the sample relative to the tip during the measurements. To obtain d*I*/d*V* spectra and maps, a lock-in amplifier with a modulation of 3–10 mV at 731 Hz was employed. AFM images were acquired using W tips placed on a qPlus tuning fork sensor [1], operating in a constant-height mode. The sensor operated in a frequency modulation mode, with a resonance frequency of approximately 27 kHz, an oscillation amplitude of about 1 Å, and a quality factor of $Q \approx 5000$. For functionalization, the tip apex was decorated with a single CO molecule by picking it up from the metal surfaces, on which the CO molecules were predeposited in situ at 5 K prior to the measurements.

TERS measurements. TERS measurements were carried out in Unisoku USM1400-LT

microscope equipped with a SPECS Nanonis controller, using Ag tips to achieve a strong plasmonic resonance in the tip-sample junction. The TERS measurements were conducted with a contact-mode, by incrementally approaching the tip closer to the specific site of the conjugated polymers on Au(111), even to a height where the tip almost contacted the sample. The initial tip height was set by the setpoint conditions, usually 0.1 V and 8 nA. Then the tip was gradually lowered with a height change step of 10 pm. The approaching distances were typically about 100~150 pm to reach the corresponding contact points. The photon collection system utilized a custom-built optical setup, as previously described [2]. Linearly polarized 532 nm laser beams with approximate powers of 0.5 mW in the contact-mode measurements were used for excitation. The laser beam was generated by a single-longitudinal-mode laser (CrystaLaser, CL532-100-SO) and fiber-coupled to the dark box. Optical alignment was achieved using two reflective mirrors. The excitation light passed through a beam splitter and entered the UHV chamber through a quartz viewport. An aspheric lens (f = 12.4 mm and NA = 0.46) focused the beam at an angle of 55 ° from the surface normal to the tip-substrate junction, resulting in a spot diameter of approximately 30 μm on the sample surface. The Raman scattered light signal was collected by the same lens, while residual laser light was filtered through a 532-nm edge filter. The Raman signal was then recorded using a CCD spectrometer (SpectraPro HRS-300, Princeton Instruments) with the slit width set at 50 μm and grating of 300 grooves/mm. CCD spectrometer integration time was 20 s.

Density functional theory calculations. The calculations of density functional theory (DFT) were implemented with the Vienna ab initio simulation package (VASP) [3]. We adopted the generalized gradient approximation (GGA) proposed by Perdew, Burke, and Ernzerhof (PBE) [4], and a van der Waals (vdW) correction scheme proposed by Grimme *et al.* (ref. [5]) was also employed. We

employed the projected augmented wave method [6, 7] with a plane-wave basis set in which the cutoff energy was set to 400 eV. The total energy convergence criterion was 1×10^{-5} eV, and during the geometrical optimizations, all the atoms except for those in the two bottom metal layers were fully relaxed until the forces on them were smaller than 0.01 eV/Å.

The Bader charge analysis approach [8, 9] was employed to evaluate the amount of charge transfer between the polymers and metal substrates. STM image simulations were based on the Tersoff–Hamann approximation [10] in the constant-current mode. For the AFM image simulations, we used a molecular mechanics model incorporating an electrostatic force approach proposed by Hapala and colleagues [11]. The effective lateral stiffness of the CO-terminated AFM tip was set to 0.5 N m^{-1}, and an effective charge of $-0.2e$ with a quadrupole (d_{z^2}) tip was used, following previous studies [12]. The oxygen atom on the tip was allowed to relax until the total force on it reached less than 10^{-4} eV $Å^{-1}$ or when the maximum of 1000 iterations was reached.

Tight-binding calculations. In real space, the tight-binding (TB) Hamiltonian of the π-electron system can be written as

$$H = t\sum_{\langle i,j\rangle} a_i^{+} a_j + t'\sum_{\langle i,j\rangle} b_i^{+} b_j + h.c.,$$

where a_i^{+} and b_i^{+} (a_j and b_j) denote the creation (annihilation) operator of a spinless electron at site i (j). The notation $\langle i,j\rangle$ indicates the nearest-neighbor hopping (NN) process. To illustrate the distinct bond orders, different parameters were chosen. The parameter t is the NN hopping integral for aromatic bonds, which was set to –3.05 eV. According the bridging bond orders obtained from DFT calculations, the hopping parameter t' was set to –5.45 and –3.45 eV for the ethynylene-bridged polypentacene (EBPP) phase and the cumulene-bridged polypentacene (CBPP) phase, respectively.

The topology of one-dimensional (1D) systems with inversion or mirror symmetry can be characterized by Z_2 invariant, which is given by

$$(-1)^{Z_2} = e^{i\sum_n \gamma_n},$$

where the sum is over the occupied bands and the Zak phase for the *n*th band (γ_n) is the Berry phase across the 1D Brillouin zone:

$$\gamma_n = i\int_{\mathrm{BZ}} dk \left\langle u_{nk} \middle| \frac{\partial u_{nk}}{\partial k} \right\rangle,$$

where k is the lattice momentum and u_{nk} is the periodic Bloch wavefunction in band n [13, 14]. Because the Zak phase depends on the choice of unit cell, the termination observed in experiments dictates the Z_2 invariant of the polymer. Base on the symmetric structure of the unit cell, there are two different choices, denoted as Unitcell-1 (Fig. S6a) and Unitcell-2 (Fig. S6d). In our work, the unit cell corresponds to the Unitcell-2, due to saturation of the terminal dangling C atom either with three hydrogen atoms or Au atoms (Fig. S6i). The Unitcell-1 possesses mirror and inversion symmetry, of which the value of Z_2 can be directly calculated. However, the Unitcell-2 does not have mirror or inversion symmetry. Based on the fact that the Z_2 invariant of the Unitcell-2 is complementary with that of the Unitcell-1, we can write their relation [15]:

$$Z_2^{\mathrm{Unitcell-2}} = 1 - Z_2^{\mathrm{Unitcell-1}}.$$

The LDOS maps of the highest occupied molecular orbital (HOMO) and lowest unoccupied molecular orbital (LUMO) are simulated by the TB wavefunction $\Psi(x, y, z_0)$ with a CO tip, considering the mixed 15% *s*-orbital and 85% *p*-orbital [16], in which tunneling amplitudes are presented by:

$$A_s(x, y) = |\Psi(x, y, z_0)|^2,$$

$$A_p(x, y) = \left|\frac{\partial\Psi(x,y,z_0)}{\partial x}\right|^2 + \left|\frac{\partial\Psi(x,y,z_0)}{\partial y}\right|^2.$$

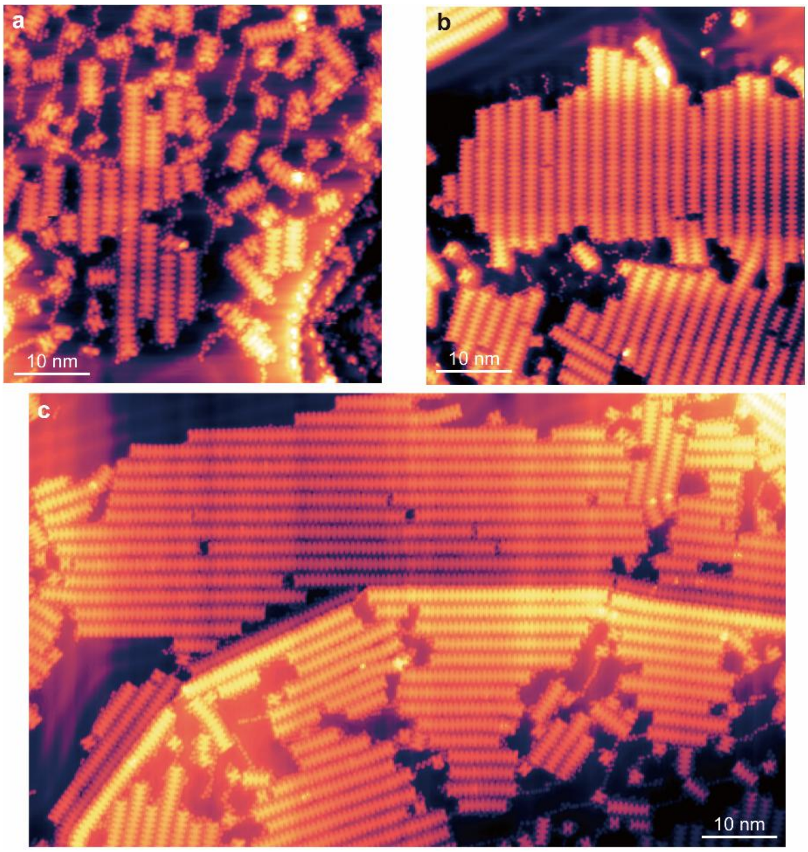

Figure S1. Large-area STM images of pentacene polymers. (a–c) Large-area STM images of pentacene polymers with various lengths after annealing at 400 K. Imaging parameters: $V_s = 1.0$ V, $I_t = 50$ pA.

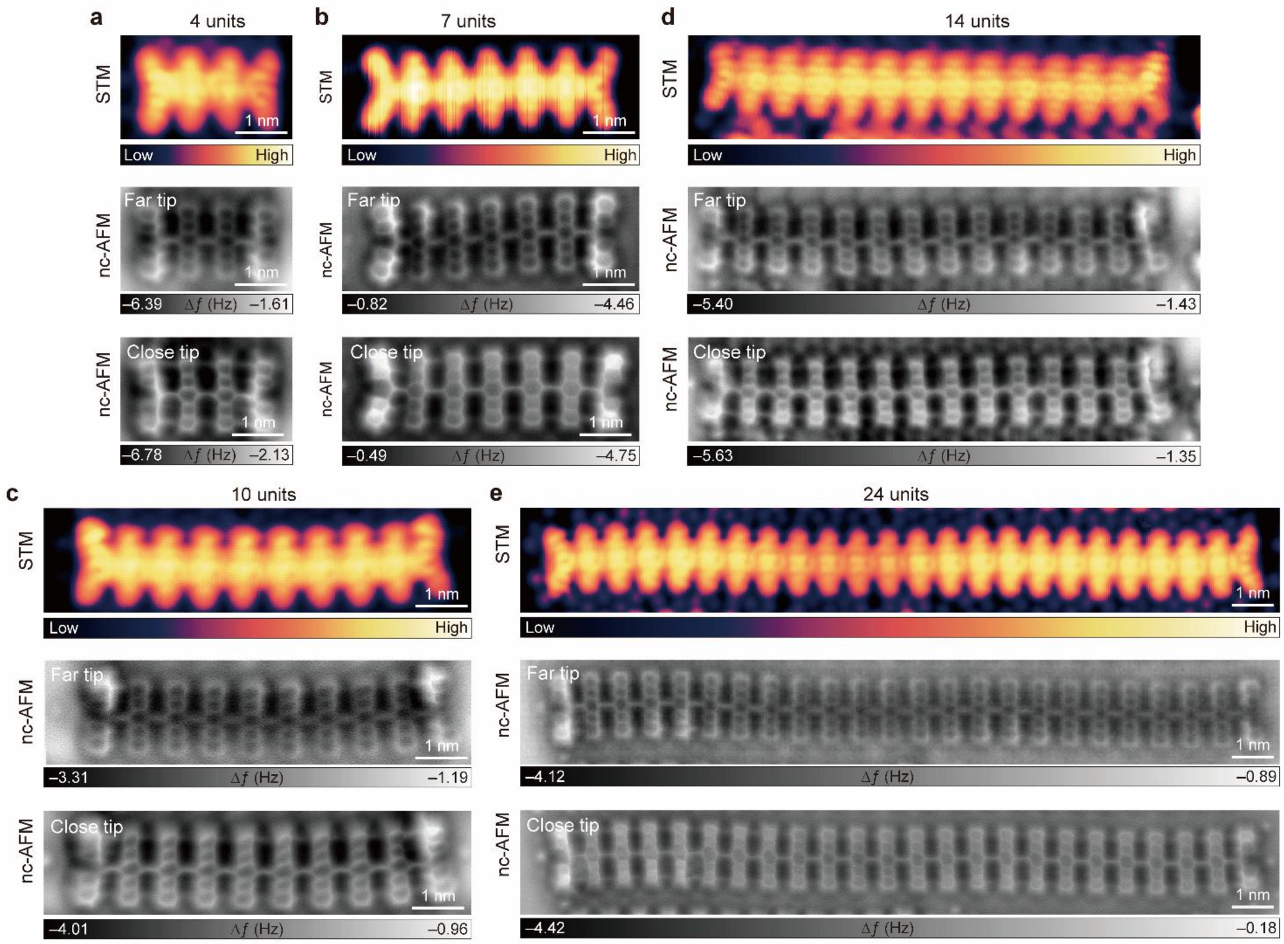

Figure S2. STM and nc-AFM images of a seven-unit-long CBPP with both termini bonding to Au surface (C–Au termination). (a–e) Experimental STM topographic images (V_s = 1.0 V, I_t = 50 pA) and laplacian-filtered nc-AFM images acquired with a CO-functionalized tip at different tip heights for n = 4 (a), 7 (b), 10 (c), 14 (d), and 24 (e). All nc-AFM images present the consistent and uniform cumulene-like character at the bridging bonds between pentacene moieties for different tip heights.

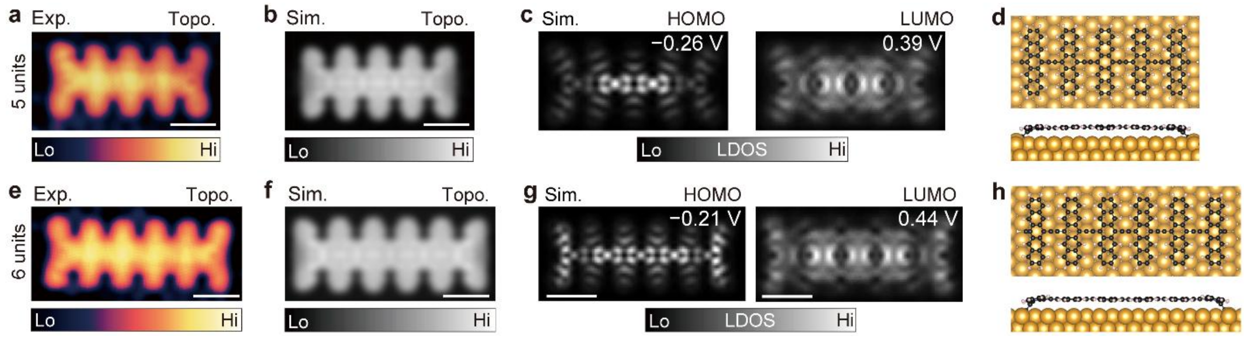

Figure S3. Experimental and simulated STM images of different species. (a and e) Experimental STM topographic images (V_s = 1.0 V, I_t = 50 pA), (b and f) simulated STM topographic images (V_s = 1.0 V) with Gaussian smoothing function by considering the tip convolution effect [17], (c and g) simulated LDOS maps of HOMO (left) and LUMO (right), and (d and h) relaxed structural models of 5-unit-long (a–d) and 6-unit-long (e–h) pentacene polymers with both C–Au termini.

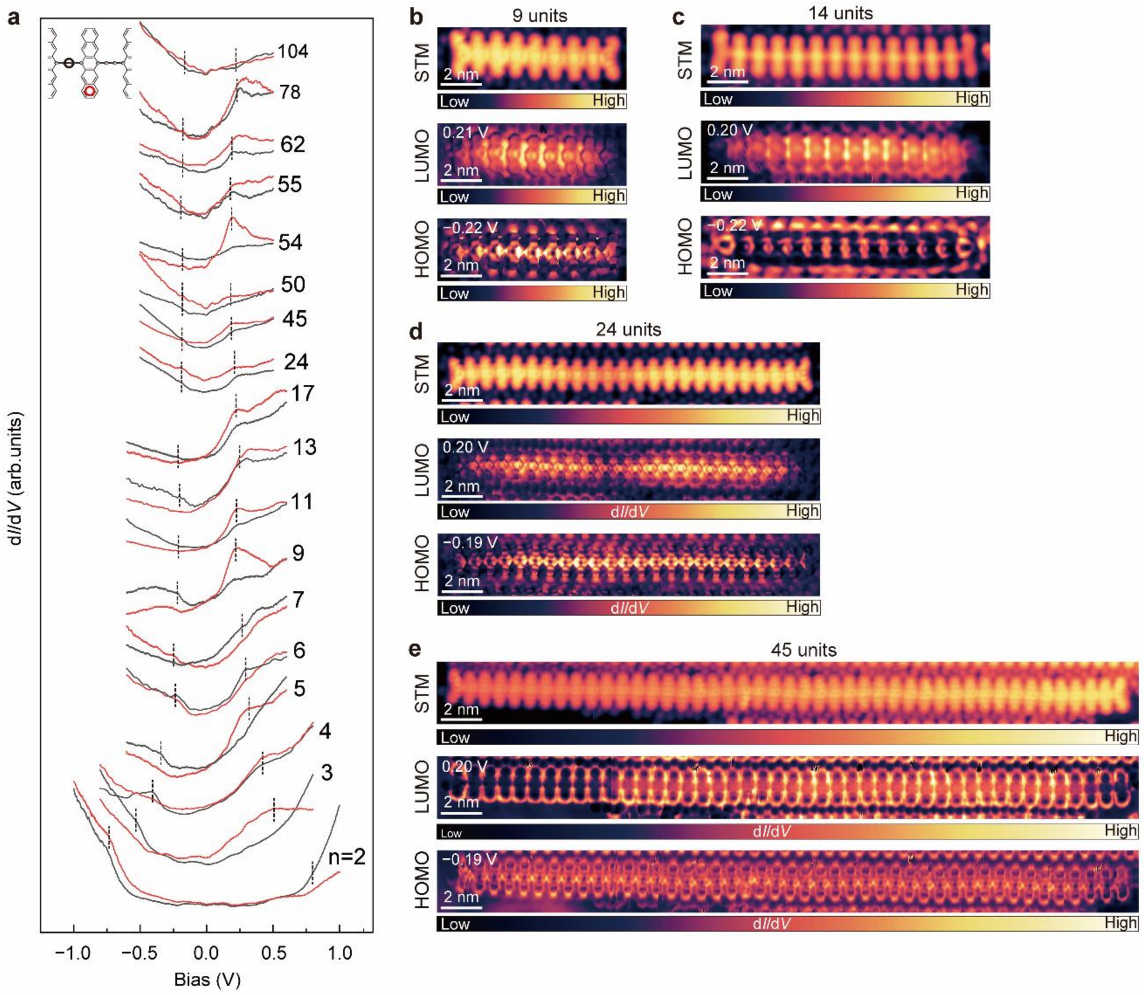

Figure S4. Additional dI/dV spectra and maps from oligomers or polymers with both termini anchored to Au. (a) Collection of typical dI/dV spectra from oligomers or polymers with labeled repeating-unit number n, vertically shifted for clarity, with the short vertical dashed lines marked the peak/step-like features corresponding to HOMO and LUMO. Spectroscopy parameters: $V_s = 1.0$ V, $I_t = 50$ pA, modulation $V_{rms} = 10$ mV for n = 2; V_s = 0.8 V, $I_t = 50$ pA, $V_{rms} = 10$ mV for n = 3 and 4; V_s = 0.5 V, $I_t = 50$ pA, $V_{rms} = 10$ mV for $n \geq 5$. (b–e) STM topographic images and dI/dV maps taken at HOMO and LUMO energies for n = 9 (b), 14 (c), 24 (d), and 45 (e), respectively. Panels (b,d,e) were taken with the CO-functionalized tip, and panel (c) was acquired with a normal tip, similar to those in Fig. S6h.

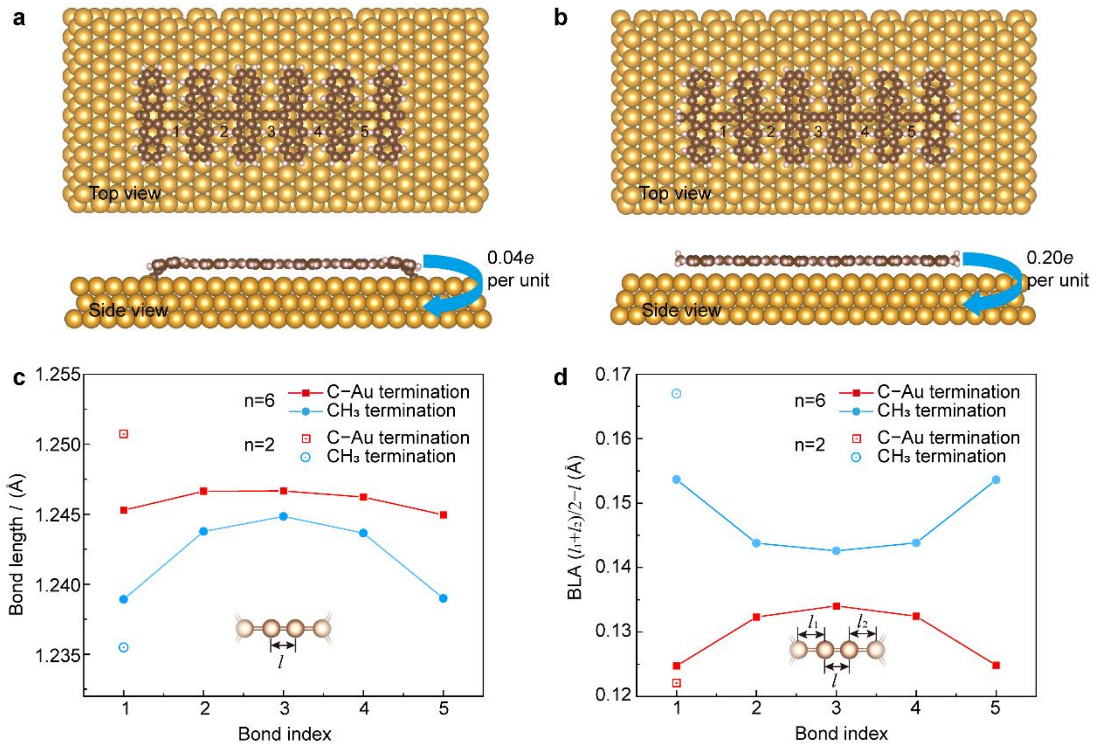

Figure S5. DFT calculations of the bond length and BLA in pentacene polymers with different terminations. (a) Relaxed top and side views of a six-unit-long polymer (n = 6) with both ends bonding to the Au surface (C–Au termination). (b) Relaxed top and side views of a six-unit-long polymer with both ends converted into CH_3 groups (CH_3 termination). The calculated amounts of charge transfer from the six-unit-long polymers to the Au surface are 0.04 (a) and 0.20 (b) electron per unit, respectively. (c) Calculated bond length l, as labeled in the inset, at the bridge bonds for n = 6 and 2. (d) Extracted BLA, calculated by $(l_1+l_2)/2–l$ as babbled in the inset, corresponding to BLA1 as defined in Fig. S7. When compared with those with CH_3 termination, the polymers with the C–Au termination present longer bridge bond lengths (c) and much smaller BLAs between the nearby bonds (d). Therefore, the negligible hole doping contributes to the cumulene-like bonds in the pentacene polymer with both ends bonding to the Au surface, while the significantly larger hole doping in the pentacene polymer with both ends converted into CH_3 groups induces the Peierls distortions and forms ethynylene-like bonds.

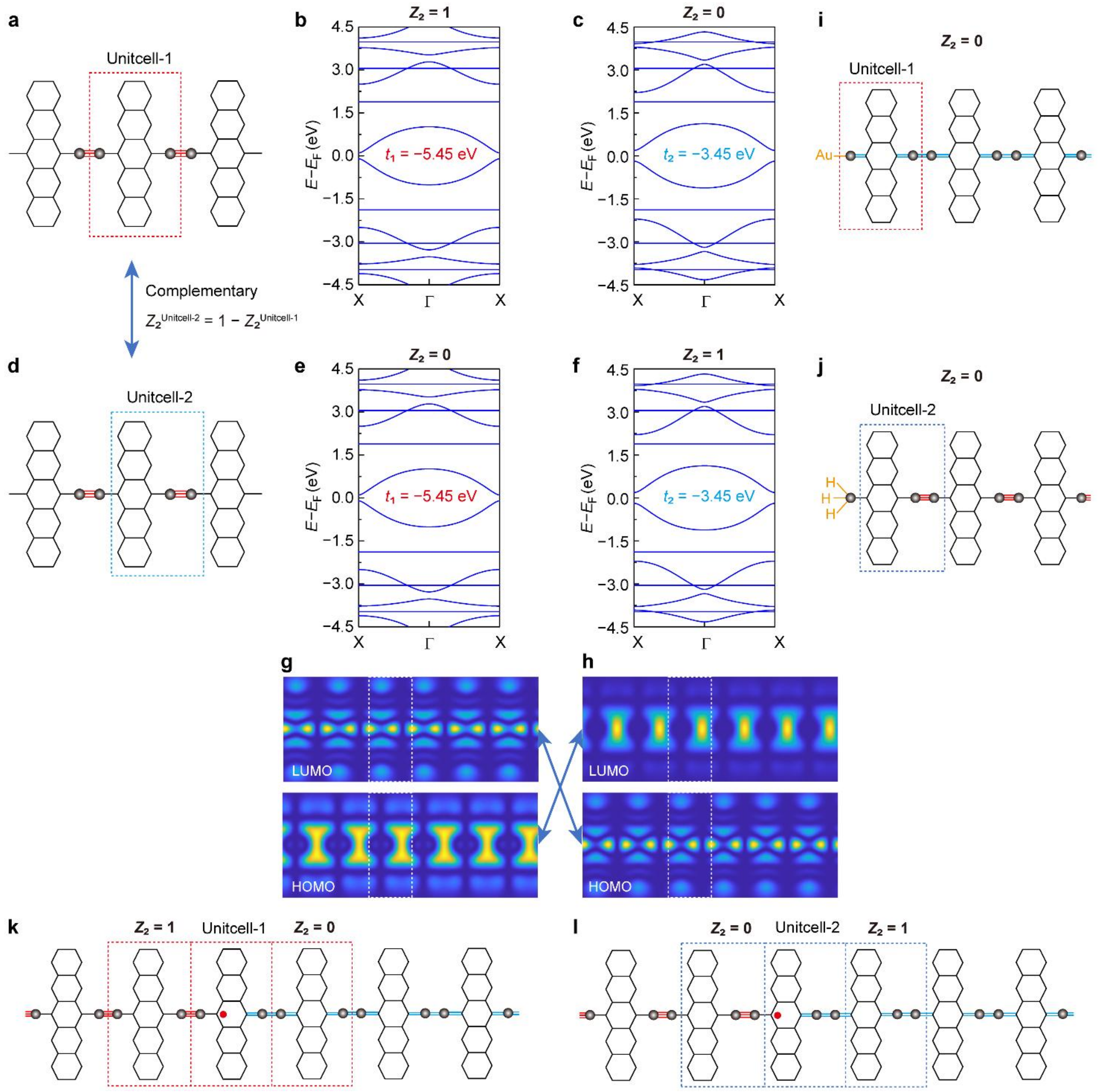

Figure S6. TB calculations of the topological properties of the pentacene polymer. (a and d) Schematics of two different unitcells (Unitcell-1 and Unitcell-2), (b and e) TB-calculated band structures with t_1 = –5.45 eV, and (c and f) with t_2 = –3.45 eV. Here t_1/ t_2 corresponds to the hopping term of the red-marked bridge bond, while other bonds with the same hopping t = –3.05 eV. In (b) and (c), Z_2 = 1 and 0 are correspondingly obtained. Since the Unitcell-2 does not have the mirror or inversion symmetry, Z_2 cannot be directly calculated. However, the Unitcell-1 and Unitcell-2 are complementary to each other [15], which can be expressed by the relation $Z_2^{\mathbf{Unitcell\text{-}2}} = 1 - Z_2^{\mathbf{Unitcell\text{-}1}}$ (see Experimental Methods for details). Then, we can get the values of Z_2 = 0 and 1 in (e) and (f), respectively. (g and h) Calculated HOMO and LUMO orbitals at the X point, corresponding to the

ethynylene-like phase in (e) and the cumulene-like phase in (f), respectively. Here a normal *s*-orbital tip is adopted. Clearly band inversion can be found, suggesting the different topologies, in line with the calculated distinct Z_2 values (e and f). (i) Schematic drawing of the unitcell with the Au-anchored termination, where the terminal carbene-like carbon contributes additional *p* electron to the π bond, resulting in the unitcell as Unitcell-1. According to (c), the CBPP chain with the Au-anchored termination is topologically trivial, with Z_2 = 0. (j) Schematic drawing of the unitcell with the CH_3 termination, where the terminal carbon is fully saturated with three H atoms and contributes no *p* electron, resulting in the unitcell as Unitcell-2. According to (e), the EBPP chain with the CH_3 termination is also topologically trivial, with Z_2 = 0. (k and l) Schematic drawings of the consistent presence of the topological soliton at the EBPP–CBPP domain wall, under the unitcell choice of both Unitcell-1 (k) and Unitcell-2 (l).

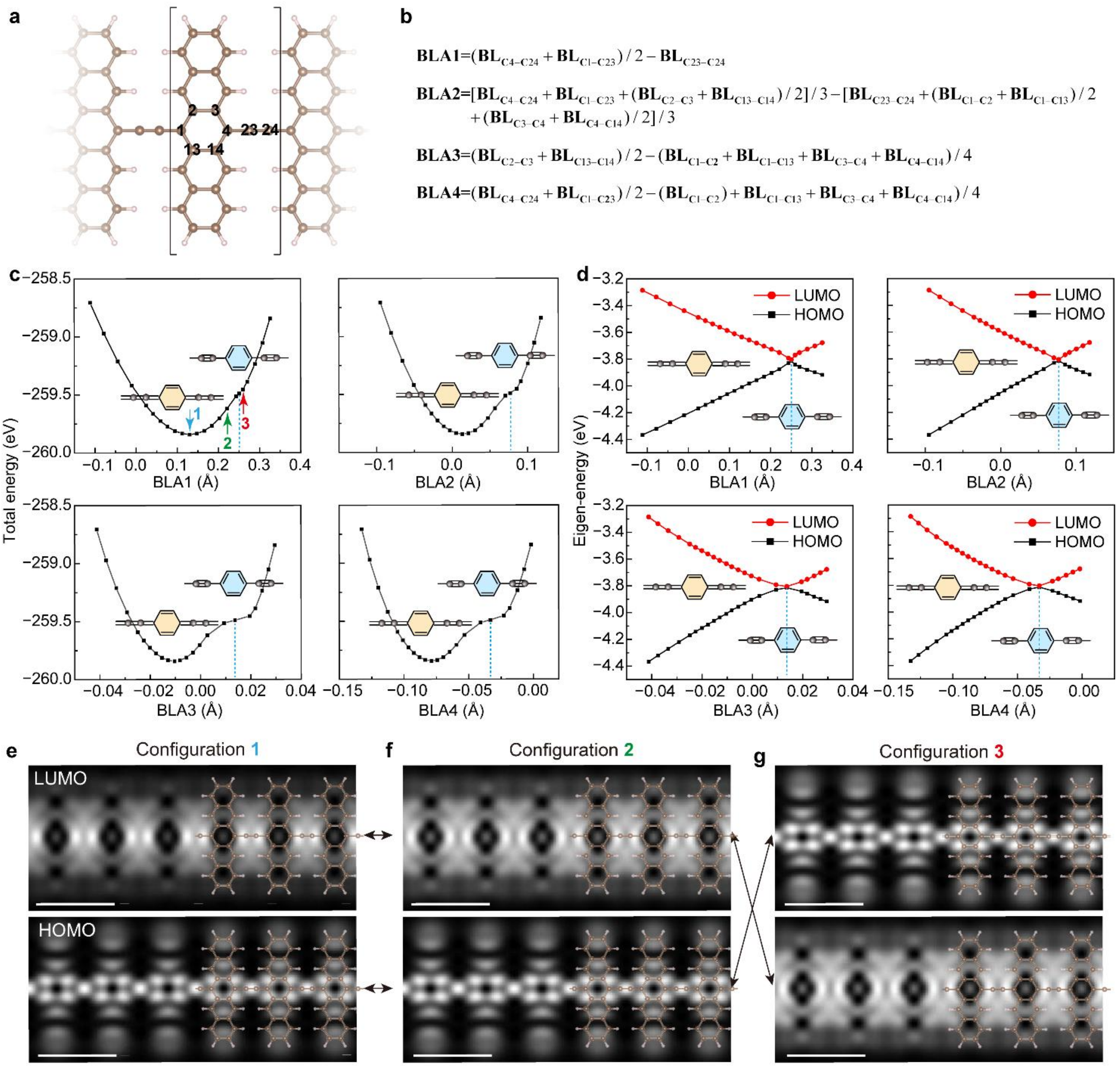

Figure S7. Calculated free energy per unitcell versus various defined BLAs. (a) Structural model labeled with the unitcell (black lines) and the C-atom numbers. (b) Four types of defined BLAs, labeled by BLA1, BLA2, BLA3, and BLA4, which take relative changes of bond length (BL) within a repeating unit as in (a). (c) Calculated total energy per repeating unit versus BLA1, BLA2, BLA3, and BLA4, which show behaviors close to the double-well potential. (d) Calculated Eigen energies of HOMO and LUMO versus order parameters BLA1, BLA2, BLA3, and BLA4. The dashed blue lines mark the reversal point of HOMO and LUMO, representing the topological phase transition. Note that in the infinite length, the ground state of the polymer is the cumulene-like phase, with the topological phase transition corresponding to the transition into the ethynylene-like phase. (e–g) Simulated LDOS maps of LUMO (top) and HOMO (bottom) of the configurations 1–3, as marked

by arrows in the top-left panel of (c). Configuration 1 is the equilibrium point, corresponding to the cumulene-like bridging bonds. Configurations 2 and 3 respectively locates at the left side (cumulene-like phase) and right side (ethynylene-like phase) of the topological phase transition critical point [dashed blue line in (c) and (d)]. Here a tip condition that contains 85% *p*-orbital and 15% *s*-orbital was used to mimic the behavior of the CO-functionalized tip. Within the same side of the point (dashed blue line) of the topological phase transition, configurations 1 and 2 display the same HOMO and LUMO patterns corresponding to the cumulene-like phase. Configuration 3 shows reversed HOMO and LUMO patterns with respect to those of configurations 1 and 2, manifesting the ethynylene-like phase. This result indicates that, based on the patterns of the HOMO and LUMO, one can determine the topological phases of the pentacene polymer.

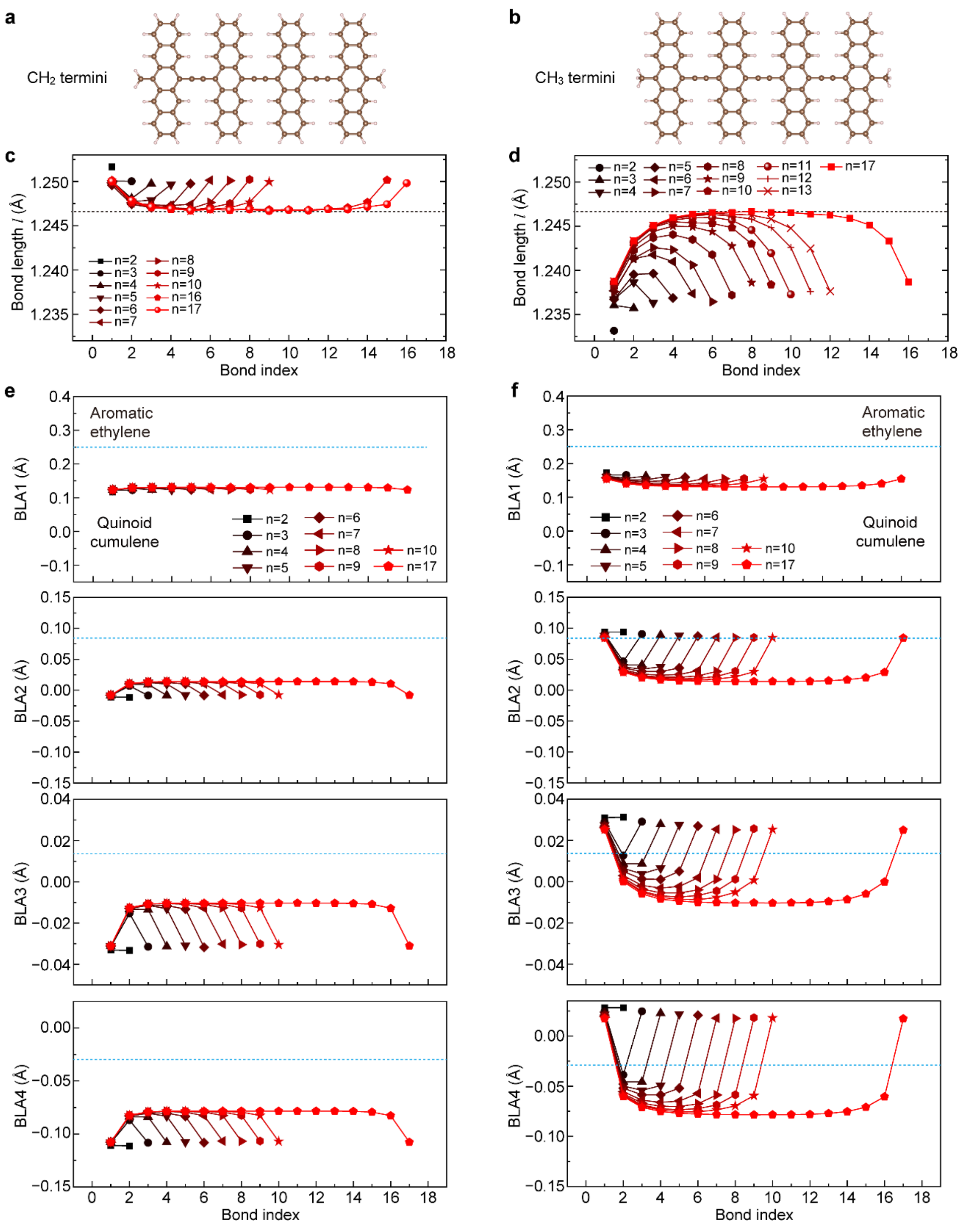

Figure S8. Calculations of the bond length and BLA in freestanding CH_2 and CH_3-terminated pentacene polymers. (a and b) Structural models of four-unit-long polymers with CH_2 and CH_3 termini, respectively. (c and d) Calculated length-dependent bond length *l* for each of the bridge bonds in the pentacene polymers with CH_3 (e) and CH_3 (f) termini, respectively. (e and f) Calculated BLA1, BLA2, BLA3, and BLA4, as defined in Fig. S7, in CH_2 (e) and CH_3 (f) terminated pentacene polymers with various length *n*. The dashed blue lines mark the phase transition critical point from

the cumulene-like phase (bottom) to the ethynylene-like phase (top), as derived from Fig. S7. In (e), the CH_2 terminated pentacene polymers present the constant cumulene-like phase independent on the polymer length, displaying the similar behavior to the observed results in pentacene polymers with C–Au termination. It suggests that we can adopt the CH_2 terminated pentacene polymers to describe the behaviors of the C–Au terminated ones observed in our experiment. In (f), the CH_3-terminated pentacene polymers present the topological phase transition from the ethylene-like to the cumulene-like phase for $n \geq 4$, especially under the order parameters BLA2, BLA3, and BLA4.

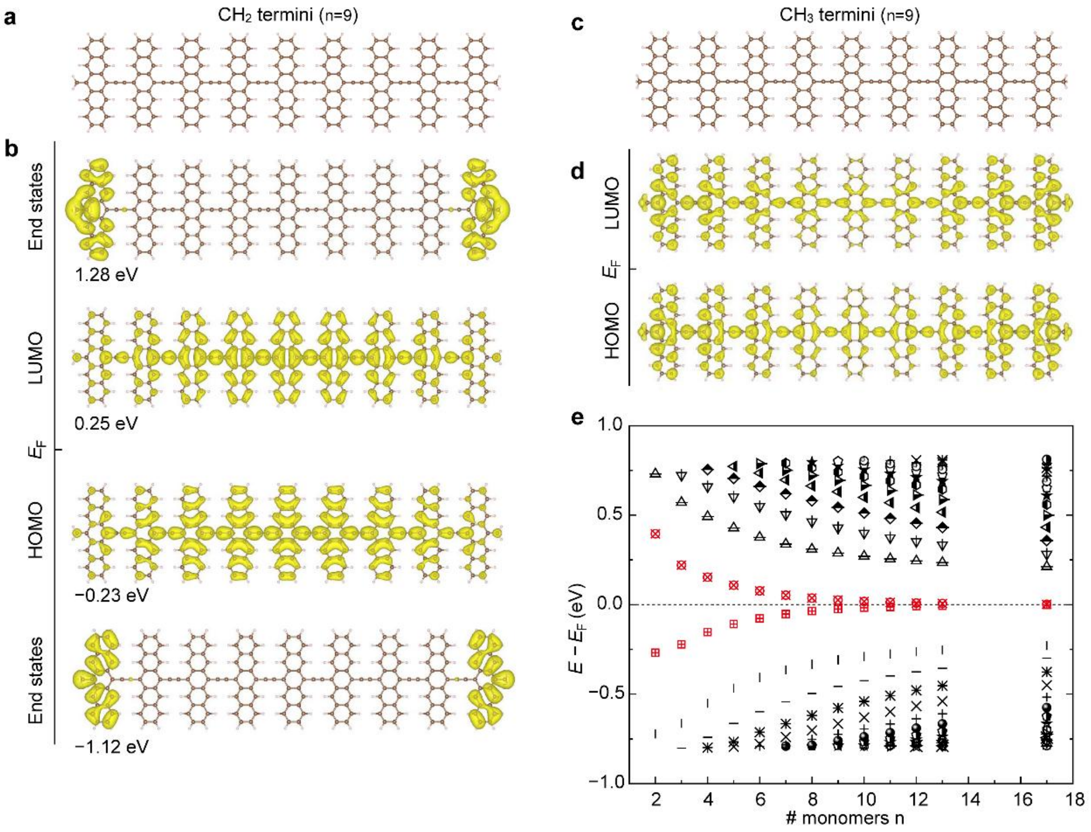

Figure S9. Calculated electronic properties of freestanding CH_2 and CH_3-terminated pentacene polymers. (a) Structural model of the CH_2-terminated polymer with n = 9. (b) Calculated charge densities of HOMO, LUMO, and end states below and above HOMO and LUMO in CH_2-terminated polymer with n = 9. These end states are doubly occupied, with no spin polarization. (c) Structural model of the CH_3-terminated pentacene polymer with n = 9. (d) Calculated charge densities corresponding to the near-zero-energy end states, corresponding HOMO and LUMO. (e) Molecular orbital evolution with the increase of n in polymers with CH_3 termini. Red marks the orbitals evolving into the zero-mode end states with the increase of n. Spin-polarized calculations suggest that no spin polarization was observed for $n \leq 3$, along with the presence of topological phase transition at $n = 4$, in line with Fig. S8.

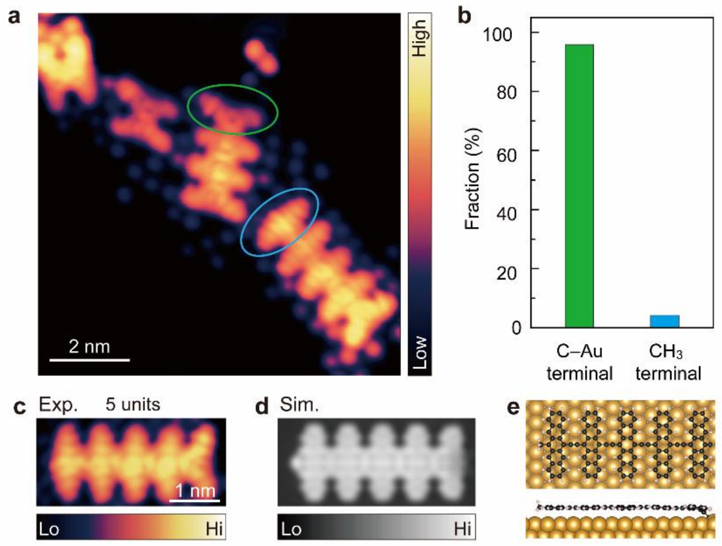

Figure S10. Statistical result of the C–Au and CH_3 terminals. (a) Experimental STM image showing the presence of the C–Au (green oval) and CH_3 (blue oval) terminals (V_s = 1.0 V, I_t = 50 pA). (b) Statistic ratios of the two distinct terminal configurations in the pentacene oligomers or polymers. The proportion of the C–Au terminal is about 95.8% and that of the CH_3 terminal is about 4.2%. While the pentacene oligomers or polymers can have two C–Au terminals, only one of the termini can be converted to the CH_3 terminal in our experiment, due to its low proportion. (c) Experimental STM topographic image (V_s = 1.0 V, I_t = 50 pA), (d) simulated STM image (V_s = 1.0 V) with Gaussian smoothing, and (f) relaxed structural model of the 5-unit-long pentacene polymers with one C–Au terminal and one CH_3 terminal.

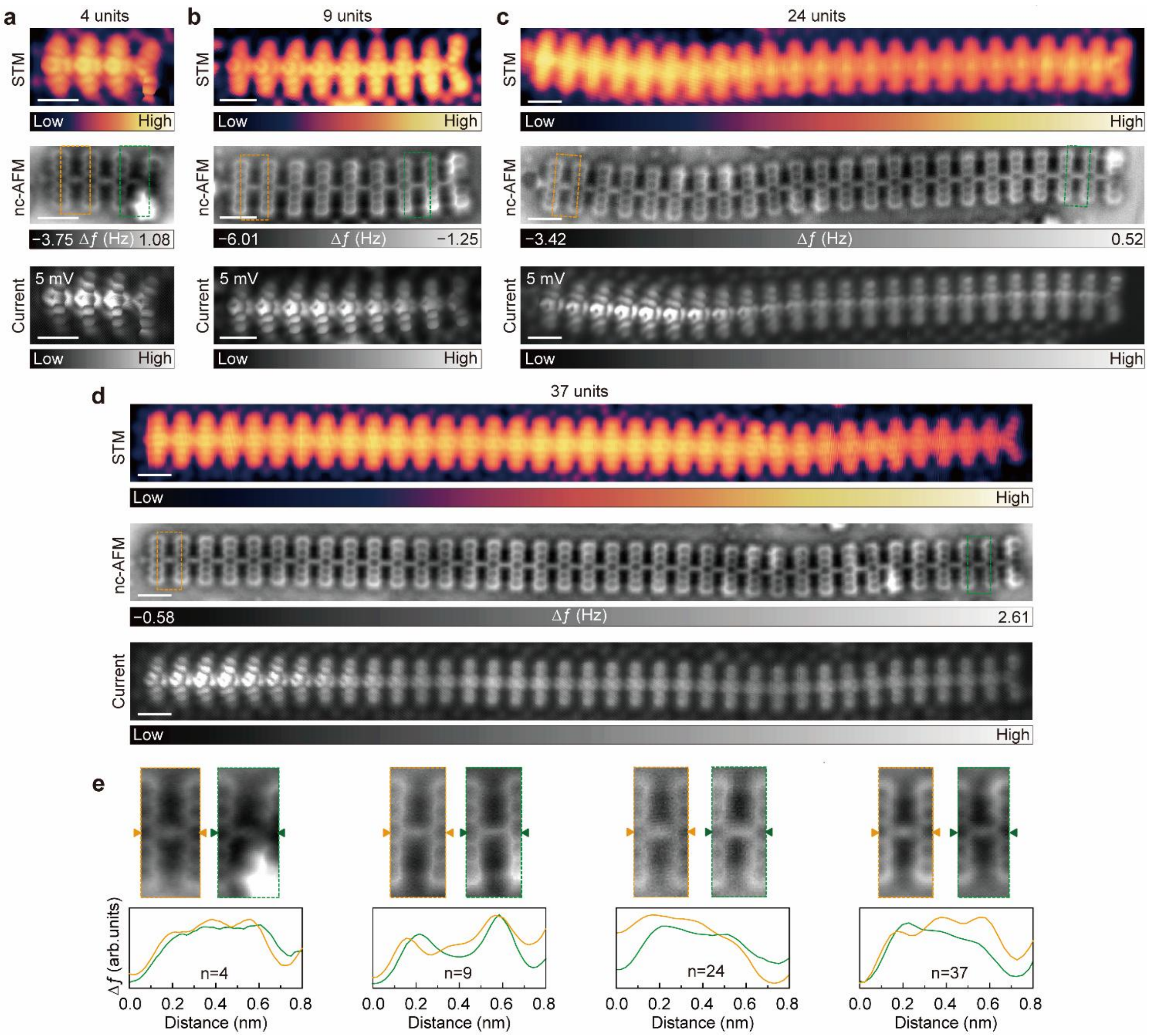

Figure S11. Additional STM and nc-AFM images of the pentacene polymers with one CH_3 terminal (left) and C–Au terminal (right) and with varying lengths. (a–d) Experimental STM topographic images (top), nc-AFM (middle) and simultaneously taken constant-height current images (bottom) of polymers with two different terminals and lengths of n = 4 (a), 9 (b), 24 (c), and 37 (d), acquired with a CO-functionalized tip. Consistent zero-modes are observed at the interfaces between the EBPP (left) and CBPP (right) segments. (e) Line profiles correspondingly along the triangle-pair marked lines in the nc-AFM images near the two terminals for n = 4 (a), 9 (b), 24 (c), and 37 (d). The orange curve taken near the CH_3 terminal display clear protrusions corresponding to the ethynylene-like bonds, while these features are unobservable in the green curve taken near the C–Au terminal, showing the signature of the cumulene-like bonds. STM imaging parameters: V_s = 1.0 V and I_t = 50 pA in (a) and

(c); V_s = 1.0 V and I_t = 30 pA in (b) and (d). AFM imaging parameters: with quality factor of $Q \approx$ 5000. During nc-AFM imaging at constant-height mode, a sample bias V_s = 10 mV was applied for taking current images simultaneously. Scale bars: 1 nm.

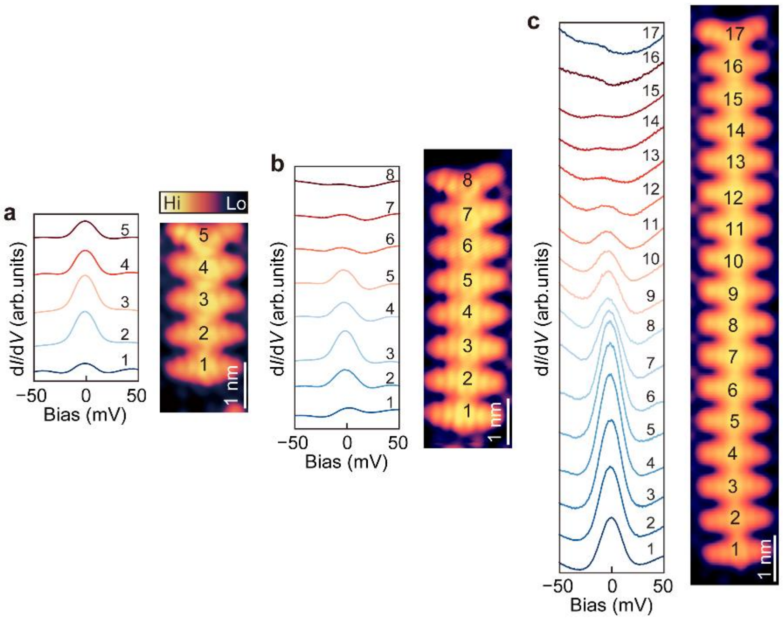

Figure S12. dI/dV spectra taken at each unit of three polymers with one CH_3 terminal (bottom) and C−Au terminal (top). (a–c) dI/dV spectra and the corresponding STM images with marked positions for the dI/dV measurements, repotted from Fig. 4a and 4d–f in the main text.

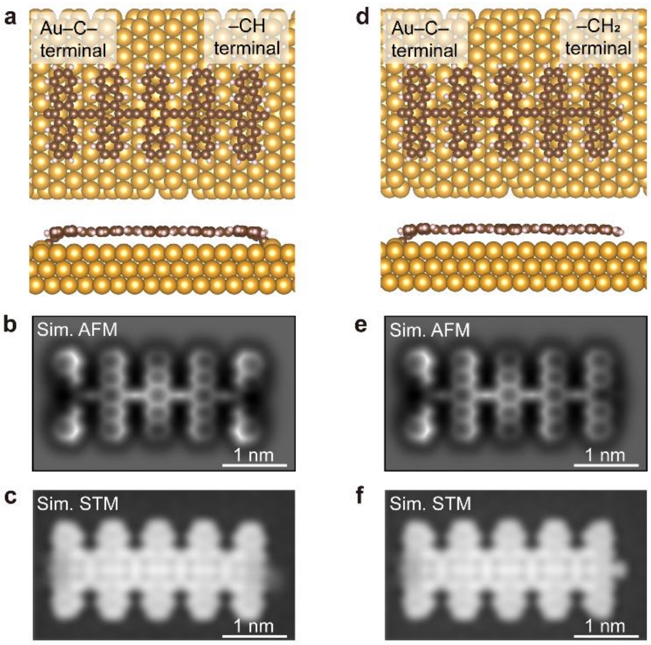

Figure S13. Simulations of the five-unit-long polymer with one C–Au terminal (left) and the other –CH or –CH_2 terminal (right). (a–c) Optimized structural model (a), simulated nc-AFM image (b), and STM image at −1 V (c) with the –CH terminal on the right. (d–f) Optimized structural model (d), simulated nc-AFM image (e), and STM image at −1 V (f) with the –CH_2 terminal on the right. As shown in the simulated AFM images (b, e), both do not present the protrusion feature as observed for the –CH_3 terminal. On the other hand, while the –CH terminal, due to the unsaturated nature, displays the quite similar feature by bending to the Au surface to that of the C–Au terminal, the simulated STM image does not clearly present the experimentally observed 'V'-shape of the end pentacene moiety.

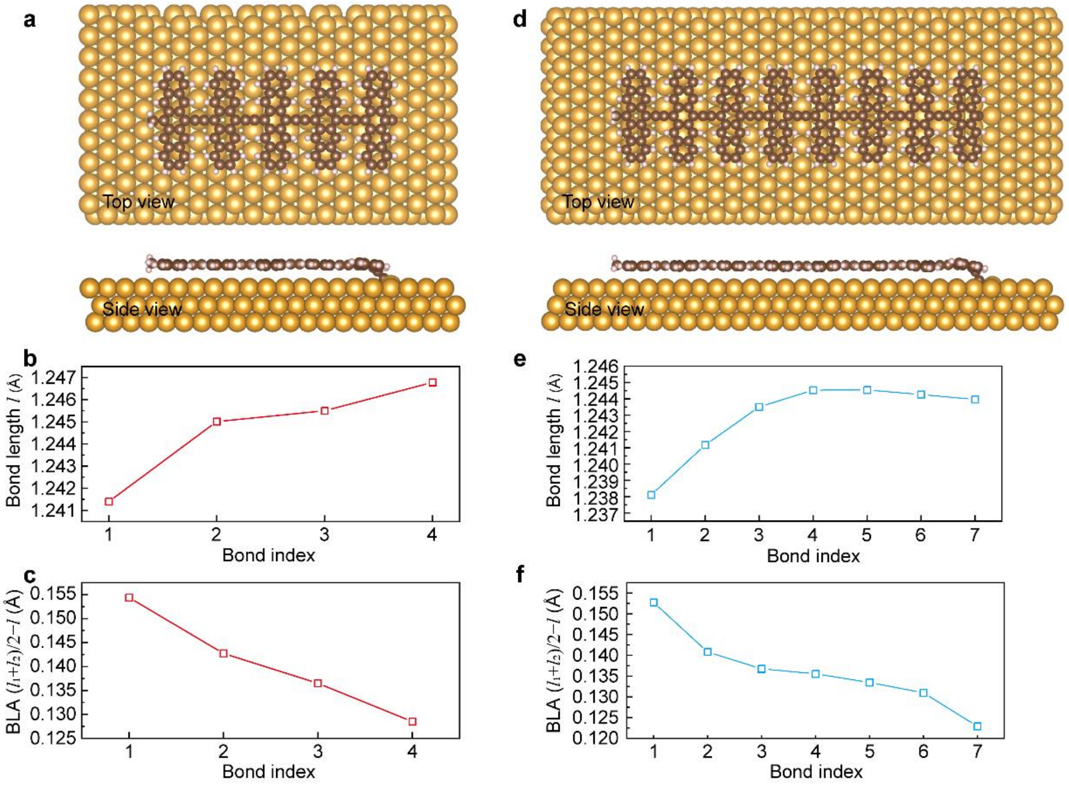

Figure S14. Calculations of the pentacene polymers with two different terminals on Au(111). (a and d) Top and side-view structural models of the pentacene polymers with one CH_3 terminal (left) and one C– Au terminal (right) for the case of n = 5 (a) and 8 (d), respectively. (b and e) Calculated bond length l of each bridge bond in (a) and (d), respectively. (c and f) Calculated BLAs, as defined in Fig. S5, for (a) and (d), respectively. Clearly shorter bond lengths and larger BLAs are observed near the CH_3 terminal, compared to those near the C–Au terminal.

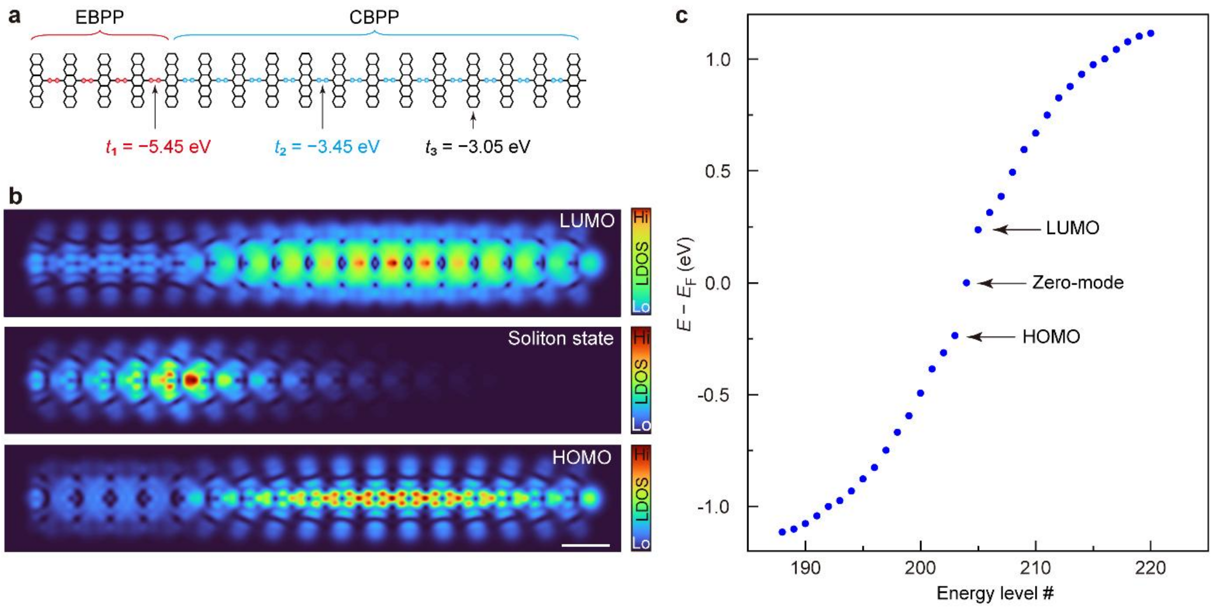

Figure S15. TB calculations of the EBPP–CBPP domain wall. (a) TB model, the same with Fig. 4l in the main text. (b) Spatial distributions of LODSs corresponding to HOMO (bottom), zero-mode soliton state (middle), and LUMO (top), simulated with a tip condition that contains 85% *p*-orbital and 15% *s*-orbital to mimic the behavior of the CO-functionalized tip. (c) Calculated energy levels based on the TB-model in (a), marked with HOMO, zero-mode soliton state, and LUMO.

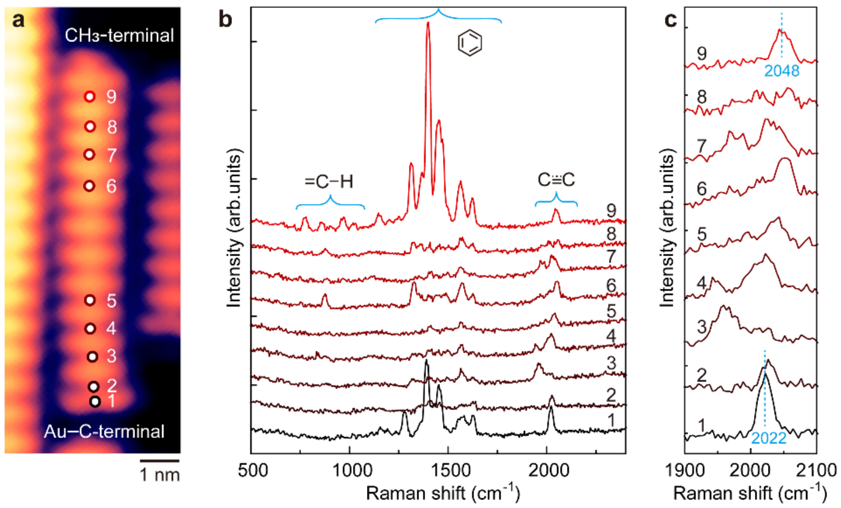

Figure S16. TERS measurement of a 12-unit-long pentacene polymer with one CH_3 terminal (top) and C–Au terminal (bottom). (a) STM topographic image of the polymer ($V_s = 1.0$ V, $I_t = 10$ pA). (b and c) TERS spectra with different frequency ranges taken at the marked sites 1–9 in (a), show multiple peaks in spectra 2–8.

References

1. Giessibl FJ. The qPlus sensor, a powerful core for the atomic force microscope. *Rev Sci Instrum* 2019; **90**(1): 011101. doi: 10.1063/1.5052264

2. Xu J, Zhu X, Tan S *et al.* Determining structural and chemical heterogeneities of surface species at the single-bond limit. *Science* 2021; **371**(6531): 818–822. doi: 10.1126/science.abd1827

3. Kresse G, Furthmüller J. Efficiency of ab-initio total energy calculations for metals and semiconductors using a plane-wave basis set. *Comput Mater Sci* 1996; **6**(1): 15–50. doi: 10.1016/0927-0256(96)00008-0

4. Perdew JP, Burke K, Ernzerhof M. Generalized gradient approximation made simple. *Phys Rev Lett* 1996; **77**(18): 3865–3868. doi: 10.1103/PhysRevLett.77.3865

5. Grimme S, Antony J, Ehrlich S *et al.* A consistent and accurate ab initio parametrization of density functional dispersion correction (DFT-D) for the 94 elements H-Pu. *J Chem Phys* 2010; **132**(15): 154104. doi: 10.1063/1.3382344

6. Blöchl PE. Projector augmented-wave method. *Phys Rev B* 1994; **50**(24): 17953–17979. doi: 10.1103/PhysRevB.50.17953

7. Kresse G, Joubert D. From ultrasoft pseudopotentials to the projector augmented-wave method. *Phys Rev B* 1999; **59**(3): 1758–1775. doi: 10.1103/PhysRevB.59.1758

8. Bader RFW. Atoms in molecules. *Acc Chem Res* 1985; **18**(1): 9-15. doi: 10.1021/ar00109a003

9. Tang W, Sanville E, Henkelman G. A grid-based Bader analysis algorithm without lattice bias. *J Phys: Condens Matter* 2009; **21**(8): 084204. doi: 10.1088/0953-8984/21/8/084204

10. Tersoff J, Hamann DR. Theory and application for the scanning tunneling microscope. *Phys Rev Lett* 1983; **50**(25): 1998–2001. doi: 10.1103/PhysRevLett.50.1998

11. Hapala P, Kichin G, Wagner C *et al.* Mechanism of high-resolution STM/AFM imaging with functionalized tips. *Phys Rev B* 2014; **90**(8): 085421. doi: 10.1103/PhysRevB.90.085421

12. Peng J, Guo J, Hapala P *et al.* Weakly perturbative imaging of interfacial water with submolecular resolution by atomic force microscopy. *Nat Commun* 2018; **9**(1): 122. doi: 10.1038/s41467-017-02635-5

13. Cao T, Zhao F, Louie SG. Topological Phases in Graphene Nanoribbons: Junction States, Spin Centers, and Quantum Spin Chains. *Phys Rev Lett* 2017; **119**(7): 076401. doi: 10.1103/PhysRevLett.119.076401

14. Zhao F, Cao T, Louie SG. Topological Phases in Graphene Nanoribbons Tuned by Electric Fields. *Phys Rev Lett* 2021; **127**(16): 166401. doi: 10.1103/PhysRevLett.127.166401

15. Cirera B, Sánchez-Grande A, de la Torre B *et al.* Tailoring topological order and π-conjugation to engineer quasi-metallic polymers. *Nat Nanotechnol* 2020; **15**(6): 437–443. doi: 10.1038/s41565-020-0668-7

16. Gross L, Moll N, Mohn F *et al.* High-Resolution Molecular Orbital Imaging Using a p-Wave STM Tip. *Phys Rev Lett* 2011; **107**(8): 086101. doi: 10.1103/PhysRevLett.107.086101

17. Wu Y, Li B, Zhu X *et al.* Polaron superlattices in n-doped single conjugated polymers. *Nat Nanotechnol* 2025; **20**(11): 1580–1587. doi: 10.1038/s41565-025-02019-7
